# A Mixed-Methods Trial of Broad Band Noise and Nature Sounds for Tinnitus Therapy: Group and Individual Responses Modeled under the Adaptation Level Theory of Tinnitus

**DOI:** 10.3389/fnagi.2017.00044

**Published:** 2017-03-09

**Authors:** Mithila Durai, Grant D. Searchfield

**Affiliations:** ^1^Eisdell Moore Centre, Section of Audiology, University of AucklandAuckland, New Zealand; ^2^Center for Brain Research, University of AucklandAuckland, New Zealand; ^3^Brain Research New ZealandAuckland, New Zealand

**Keywords:** clinical trial, tinnitus, auditory perception, adaptation, psychoacoustics, ecology model, sound therapy

## Abstract

**Objectives:** A randomized cross-over trial in 18 participants tested the hypothesis that nature sounds, with unpredictable temporal characteristics and high valence would yield greater improvement in tinnitus than constant, emotionally neutral broadband noise.

**Study Design:** The primary outcome measure was the Tinnitus Functional Index (TFI). Secondary measures were: loudness and annoyance ratings, loudness level matches, minimum masking levels, positive and negative emotionality, attention reaction and discrimination time, anxiety, depression and stress. Each sound was administered using MP3 players with earbuds for 8 continuous weeks, with a 3 week wash-out period before crossing over to the other treatment sound. Measurements were undertaken for each arm at sound fitting, 4 and 8 weeks after administration. Qualitative interviews were conducted at each of these appointments.

**Results:** From a baseline TFI score of 41.3, sound therapy resulted in TFI scores at 8 weeks of 35.6; broadband noise resulted in significantly greater reduction (8.2 points) after 8 weeks of sound therapy use than nature sounds (3.2 points). The positive effect of sound on tinnitus was supported by secondary outcome measures of tinnitus, emotion, attention, and psychological state, but not interviews. Tinnitus loudness level match was higher for BBN at 8 weeks; while there was little change in loudness level matches for nature sounds. There was no change in minimum masking levels following sound therapy administration. Self-reported preference for one sound over another did not correlate with changes in tinnitus.

**Conclusions:** Modeled under an adaptation level theory framework of tinnitus perception, the results indicate that the introduction of broadband noise shifts internal adaptation level weighting away from the tinnitus signal, reducing tinnitus magnitude. Nature sounds may modify the affective components of tinnitus via a secondary, residual pathway, but this appears to be less important for sound effectiveness. The different rates of adaptation to broadband noise and nature sound by the auditory system may explain the different tinnitus loudness level matches. In addition to group effects there also appears to be a great deal of individual variation. A sound therapy framework based on adaptation level theory is proposed that accounts for individual variation in preference and response to sound.

**Clinical Trial Registration:** www.anzctr.org.au, identifier #12616000742471.

## Introduction

Subjective tinnitus is the involuntary perception of one or more sounds by an individual, in the absence of an external physical source (Henry et al., 2005; Moller, 2006; Kaltenbach, 2011; De Ridder et al., 2014). It is now broadly understood to arise as a result of peripheral lesions in the auditory system resulting in altered cortical input. This triggers compensatory neuroplasticity changes across several overlapping brain networks (Schecklmann et al., 2013a,b; Vanneste et al., 2011, 2013; Husain and Schmidt, 2014). Final tinnitus magnitude is thought to result from differences in personality and activity within auditory, emotion, attention, and memory networks (Searchfield et al., 2012; Searchfield, 2014; Durai et al., 2015). Fifteen to twenty percentage of the tinnitus population experience significant disruption to quality of life (Heller, 2003; Hoffmann and Reed, 2004), manifesting as impaired concentration, problems with hearing, irritation, frustration and annoyance, anxiety, depression, disruption of everyday activities, and disturbed sleep (Davis and El Refaie, 2000; Heller, 2003; Bartels et al., 2010; Malouff et al., 2011). Reports of tinnitus affect vary a great deal from individual to individual, leading to models of tinnitus that include individual psychology and personality as strong contributors (Searchfield et al., 2012; Searchfield, 2014). A failure to account for the heterogeneous nature of tinnitus has likely contributed to the difficulties in identifying useful therapies.

Sound therapy is currently used in several tinnitus treatment paradigms. Sound therapy uses external sounds to modify tinnitus perception and/or reactions to it (Scott et al., 1990; Jastreboff, 1999; Henry et al., 2006; Tyler, 2006; Hoare et al., 2011; Searchfield et al., 2012). Immediate effects are provided by masking (Scott et al., 1990; Tyler, 2006), and long-term changes in tinnitus functional networks have also been observed (Noreña and Eggermont, 2006; Távora-Vieira et al., 2011; Tyler et al., 2012). The potential for tinnitus and external sound to interact exists as both undergo similar auditory processing within the system, including feature extraction, schema formation, and semantic objective formation (Searchfield et al., 2012; Searchfield, 2014). Although categorization of patient characteristics has been used to guide focus of treatments [e.g., hearing aids, counseling, use of sound therapy (Jastreboff, 1998)] and some sound therapies alter the therapeutic sound based on pitch (Stein et al., 2016) and give participants choices about the stimuli, how sound is selected based on individual needs does not appear to be widespread or documented. Sounds used in therapy include broadband noise (BBN), narrow-band noise (either pitch-matched or unmatched to tinnitus), nature sounds or music (Sandlin and Olsson, 1999; Vernon and Meikle, 2000; Folmer and Carroll, 2006). Despite its popularity, there is no consensus as to the most appropriate sound parameters for tinnitus therapy, or if the treatment provides independent benefit over psychological effects (Tyler, 2006; McKenna and Irwin, 2008; Hobson et al., 2010) or hearing aids (Henry et al., 2015). Several recent studies using different types of sound have shown small (Kim et al., 2014) or no significant differences in effect (Barozzi et al., 2016) between different therapy sounds on tinnitus. There is some evidence that dynamic sounds that temporally vary may provide greater benefit for reducing tinnitus symptoms compared to fixed intensity sounds (Vernon and Meikle, 2000; Henry et al., 2004; Davis, 2006; Hann et al., 2008). Customized music and counseling applied via the Neuromonics Tinnitus Treatment for 6 months resulted in greater alleviation of tinnitus symptoms and greater user acceptability than when participants were provided with counseling and BBN, or counseling only (Davis et al., 2008). Schreitmüller et al. (2013) observed that nature sounds, even though they presented with higher dynamics and higher masking thresholds, were accepted more by the listener than white noise. Ocean or wave sounds have recently been introduced by several hearing aid manufacturers in their tinnitus therapy devices (Callaway, 2014; Dos Santos and Powers, 2015).

The reasons why temporally varying sound may be more effective in treating tinnitus in some individuals are unclear. The added therapeutic success of dynamic sounds, particularly sounds relevant to an individual's everyday environment, may be due to the provision of greater informational (central) auditory masking, whereby both therapeutic sound and tinnitus compete for cognitive resources (Kidd et al., 2002). Informational or “central” masking is possible with tinnitus, as the phenomenon is due to central processing itself. Another way in which music or nature sounds can promote relief is by engaging the emotional regions of the brain; as relaxation aids (Davis et al., 2008; Hanley and Davis, 2008). Unpleasant sounds mimicking tinnitus have been found to activate the tinnitus network more strongly than neutral tones (Schlee et al., 2008). Simulation of tinnitus (using an aversive tinnitus-like auditory stimuli) in patients without tinnitus have been shown to activate neural networks comparable to that of tinnitus, including recruitment of the limbic system (Mirz et al., 2000a,b). Differences in processing of pleasant sounds have also been observed between tinnitus patients compared to those without hearing loss or tinnitus (Carpenter-Thompson et al., 2014), as greater activation of the bilateral hippocampus and right insula. It is possible that tinnitus and emotionally negative auditory perceptions from known sources may share similar neural processing networks, which are counteracted by the presence of pleasant stimuli. Short term exposure to emotional stimuli in the auditory modality (but not visual modality) influences ratings of tinnitus: with presentation of more unpleasant sounds resulting in increased tinnitus magnitude (Durai et al., 2017b).

An ecological model (Searchfield, 2014) of tinnitus that incorporates Adaptation Level Theory (ALT; Helson, 1964; Searchfield et al., 2012) has been proposed to account for individual differences in responses to sound therapy, in which a multitude of inherent, and environmental factors interact to determine final tinnitus magnitude. The ALT is based on Helson's (Helson, 1948, 1964) theory, whereby an adaptation level (AL) acts as an internal anchor/reference point used to make sensory magnitude estimations, and this is susceptible to change over time and context (Helson, 1948, 1964; Coren and Ward, 1989). For loud and/or annoying tinnitus, a high internal AL is established—thus the tinnitus is perceived as being of high magnitude. The final AL magnitude estimates of tinnitus, as well as distress judgements, are derived by interactions between the focal component (tinnitus), contextual component (any background noise or applied sounds), and various residual components (individual cognitive and behavioral characteristics such as personality traits, memory, and past experiences, emotion, etc.) (Searchfield et al., 2012; Searchfield, 2014; Durai et al., 2015). The ALT model of tinnitus predicts that BBN and sounds that fluctuate or are emotive (such as nature sounds in our soundscape) should both affect tinnitus positively but through different mechanisms. Variables affecting the success of different sounds might include individual-specific top-down processing related to personality, memory, prediction, attention, and emotion as well as bottom up processes related to primitive auditory analysis such as contrast (Searchfield et al., 2012). Up until this study there have been no controlled trials to test sound therapy based on the ALT model. The presence of several influencing factors on tinnitus-external sound interactions might account for individual success (or lack of success) with sound therapy. A successful sound therapy is not one that affects tinnitus alone; it must be comfortable as well. Testing different parameters and individual preferences of sound therapy are therefore significant in strengthening support for, and improving, sound therapy effectiveness (Barros Suzuki et al., 2016).

We hypothesized that nature sounds would affect top-down processing, and this, along with positive effects on emotion would result in greater reduction in tinnitus magnitude than BBN, that would primarily affect bottom-up processing. Barozzi et al. (2016) found that nature and BBN resulted in similar reductions of the Tinnitus Handicap Questionnaire (THQ) following 6 months of administration, but they did not explore individual characteristics and mechanism of benefit relative to study outcomes. An experimental study piloting some of the methods employed here (Durai et al., unpublished manuscript) found that 30 min administration of unpredictable surf-like sound resulted in significantly lower tinnitus loudness than a predictable surf sound. A 2 week feasibility trial found greater number of participants preferred the unpredictable surf sound (Durai et al., unpublished manuscript). The effects of other contributory factors (e.g., greater relaxation to one sound over the other, different emotions evoked by the two sounds, anticipation) were not controlled for in that short-term trial. A longer-term clinical trial comparing BBN and nature sounds measuring various individual residuals (e.g., emotion, attention) was deemed critical to understand sound therapy effects.

## Methods

This study was approved by the University of Auckland Human Participants Ethics Committee. All participants gave written informed consent in accordance with the Declaration of Helsinki. This trial was retrospectively registered on Australian New Zealand Clinical Trials Registry (ANZCTR; Trial #12616000742471).

### Trial design

**Figure 1:**
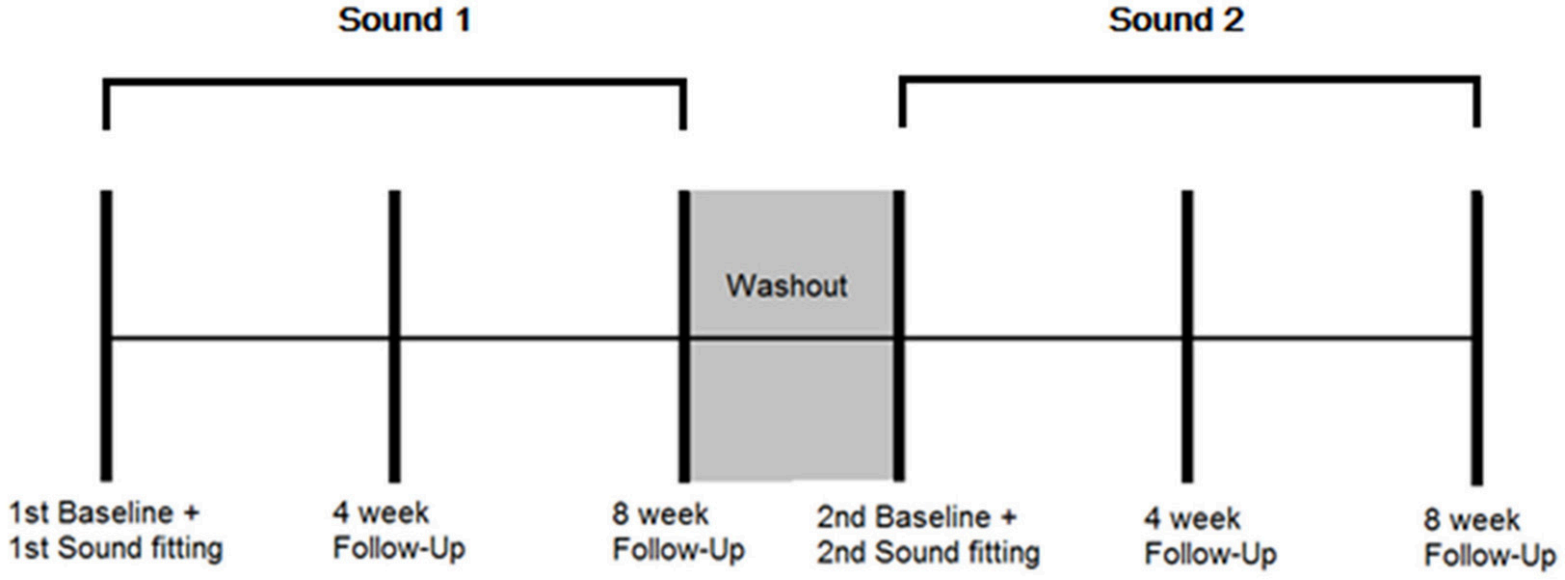
**Protocol for data collection**. Multiple outcome measurements were taken at the following time points: 1st sound fitting (Baseline), and 4 and 8 weeks after first fitting while the sound was being used. A washout period of 3 weeks followed in which no sound was administered. Multiple outcome measurements were then taken at the following time points: 2st sound fitting (Baseline), and 4 and 8 weeks after second fitting while the sound was being used.

A randomized controlled, cross-over study design using mixed (qualitative and quantitative) methods was employed. Repeated outcome measures were obtained at three time points: baseline when the sound was first fitted, 4 weeks after administration, and 8 weeks after administration for both BBN and nature sound therapies. There was a 3 week wash-out period in between the two conditions. The outcome measures taken at each appointment and time-frame protocol for data collection are presented in Table 1 and Figure 1, respectively.

**Table 1:** **Outcome measurements taken at the different time points of the trial**.

	**1st baseline + 1st sound fitting**	**4 week follow-up**	**8 week follow-up**	**2nd baseline + 2nd sound fitting**	**4 week follow-up**	**8 week follow-up**
Quantitative questionnaires	TFI	TFI	TFI	TFI	TFI	TFI
	Tinnitus loudness rating (1–10)	Tinnitus loudness rating (1–10)	Tinnitus loudness rating (1–10)	Tinnitus loudness rating (1–10)	Tinnitus loudness rating (1–10)	Tinnitus loudness rating (1–10)
	Tinnitus annoyance rating (1–10)	Tinnitus annoyance rating (1–10)	Tinnitus annoyance rating (1–10)	Tinnitus annoyance rating (1–10)	Tinnitus annoyance rating (1–10)	Tinnitus annoyance rating (1–10)
	PANAS	PANAS	PANAS	PANAS	PANAS	PANAS
	DASS	DASS	DASS	DASS	DASS	DASS
Psychoacoustic measurements	LLM	LLM	LLM	LLM	LLM	LLM
	MML	MML	MML	MML	MML	MML
Attention measurements	CAB		CAB	CAB		CAB
Qualitative		Qualitative interview schedule	Qualitative interview schedule		Qualitative interview schedule	Qualitative interview schedule

### Participants

The inclusion criteria were: adults aged between 18 and 69 years residing in the Auckland region (NZ), constant tinnitus and a minimum weighted score of 21 on the Tinnitus Functional Index (TFI; this cut-off score is calculated based on convergent validity results between TFI mean scores and response levels of a tinnitus global severity item; a score of 21 delineates individuals who consider their tinnitus as problematic from those who do not view tinnitus as a problem; Meikle et al., 2012), normal middle ear function, and a maximum of a moderate degree of hearing loss (<70 dB loss on average across frequencies). A participant information sheet was provided to participants that outlined the background and aims of the trial and details of measurements to be taken at various appointments.

#### Initial assessments

Following a comprehensive case history [Tinnitus Case History Questionnaire; TCHQ; (Langguth et al., 2007)], a hearing assessment was conducted in a sound treated room (ISO 8253–1:2010). Pure tone audiometry (0.25–16 kHz, Carhart and Jerger, 1959) was undertaken using a GSI-61 two-channel audiometer and TDH-50P headphones or E.A.RTONE 3A insert earphones and Sennheiser HDA-200 high-frequency headphones. Tympanometry was undertaken using a GSI Immittance audiometer to check middle-ear function. Tinnitus pitch match was carried out using tinnitus testing software (The University of Auckland) using high frequency circumaural headphones (Sennheiser HDA-200). Tinnitus pitch match was assessed throughout the test frequency range of 0.25–16 kHz using a two-alternative forced-choice (2AFC) method. Each tone was presented at a sensation level of 15 dB SL. Pitch match was then compared to tones one octave above and below to rule out octave confusion. The measurement was repeated until two repeatable responses were obtained. The Multidimensional Personality Questionnaire (MPQ; Tellegen, 1982) was also administered at the initial appointment to measure levels of individual personality traits.

### Interventions

#### Sound therapy stimuli

Broadband noise (BBN) was generated using Audacity 2.1.2. (A.2.1.2., 2016). The natural sounds were Surf, Cicadas/Farm Sounds and Rain sounds directly recorded from the natural setting by the researchers using a Roland R-05 WAV/MP3 Recorder with CS-10 EM binaural ear level microphones and edited to 30 min duration using Audacity 2.1.2. software (A.2.1.2., 2016). All stimuli were adjusted for sound level such that the long-term average loudness (dB SPL) was equivalent. The detailed acoustic parameters and spectrum of each sound stimulus are provided in Appendix A in Supplementary Material. Each sound therapy was administered for 8 weeks each via a Philips ViBE SA4VBE08KF/97 4GB MP3 Player and Panasonic RP-HJE290GUK Premium Black Earphones with a Budloks Earphone Sports Grip earpiece attached for secure retention within the ear. Participants were instructed to listen to the sound therapy for a minimum of 1 h per day.

#### Tinnitus loudness and annoyance functions and selection of nature sound

BBN and the three nature sounds were played for 2 min each (in randomized order) at the participants desired comfort level. At the end of each sound, participants were asked to rate the sound on a scale of 1–10, with 1 corresponding to a highly negative and/or unpleasant sound and 10 corresponding to highly positive and/or pleasant sound.

BBN and the three nature sounds again were played (in randomized order) to participants at increasing sound levels: from the threshold at which the sound was first heard to the minimum masking level (MML) where the sound first masked the individual's tinnitus. Tinnitus annoyance, tinnitus loudness ratings and noise annoyance ratings (on a scale of low 1–10 high) were undertaken at fixed sound level intervals from 0 dB SL to MML. Participants were also asked to judge the relative loudness of tinnitus and noise on a scale of 1–10 as each sound was increased in sound level, with 1 corresponding with the nature noise being not audible (tinnitus is only audible) and 10 being tinnitus is not audible (fully masked by the sound).

At the end of the task, participants selected which nature sound stimuli they preferred to use in the trial, and this was administered as the Nature sound intervention. Participants were also asked the following questions:

1. Why did you select this particular sound?
2. What kind of feelings (if any) does this sound elicit?

The average valence rating, Equal Loudness Level (the sound level where the combined tinnitus and noise loudness rating given to a noise level was 5, indicating that both tinnitus and noise were of equal perceived loudness) and Equal Annoyance Level (the sound level tinnitus at which annoyance rating functions and noise annoyance rating functions intersect, indicating that both tinnitus and noise are of equal perceived annoyance) of all participants were calculated and recorded for BBN and each environmental sound.

The MP3 volume was initially set for BBN and the nature sound to be one step (10%) below Equal Loudness Level, and if participants preferred it to be slightly higher or lower due to comfort reasons, the sounds were further adjusted accordingly. The final sound set was therefore at an audibility where sound interfered with tinnitus perception but that was also comfortable for the user.

### Outcomes

#### Assessments: questionnaires for clinical evaluation

The Tinnitus Functional Index (TFI; Meikle et al., 2012) was the primary outcome measure, in addition, the following outcome questionnaires were used: Tinnitus Loudness Rating (scale of 1–10), Tinnitus Annoyance Rating (scale of 1–10), Positive and Negative Affect Schedule (PANAS; Tellegen, 1982; Watson et al., 1988), and the Depression, Anxiety and Stress Scale (DASS Scale; Lovibond and Lovibond, 1995). The TFI (Meikle et al., 2012) is a recently developed questionnaire and assesses both severity of tinnitus and its impact on life over eight diverse subscales of intrusiveness, sense of control, cognitive, sleep, auditory, relaxation, quality of life, and emotional. TFI shows high responsiveness to treatment-related change and has been validated as an intake questionnaire with good test-retest reliability in the NZ population (Chandra et al., 2014). Tinnitus loudness ratings were made on a 10-point rating scale where 1 corresponded to a very quiet and 10 with extremely loud. Annoyance ratings were made on a similar scale with one being very low in distress and/or annoyance and 10 being extremely high in distress and/or annoyance. PANAS measures the extent to which positive and negative emotional states are experienced by an individual over the period of the past week. The DASS scale measures levels of affective symptoms.

#### Assessments: psychoacoustic tinnitus characteristics

Tinnitus psychoacoustic outcomes were measured using tinnitus testing software (The University of Auckland) using high frequency circumaural headphones (Sennheiser HDA-200). Loudness level matching (LLM) was obtained using the pitch-matched stimulus sound at 30 dB above the threshold level and decreasing it slowly in 2 dB steps until the participant stated it was same loudness as their tinnitus. This was repeated three times, and the average of the last two runs was taken. This was subtracted from the threshold level to obtain a level match in dB SL. Minimum masking level (in dB SL) was obtained using a narrow-band noise (NBN) stimulus of 1/3 octave width, raising it from the threshold level until the participant reported that the tinnitus was no longer audible. This procedure was repeated three times, and the average level was calculated. This was subtracted from the threshold level to give the MML match in dB SL.

#### Assessments: attention

The Comprehensive Attention Battery (CAB®; Rodenbough, 2003) was used to behaviorally measure individual attention and concentration ability. The CAB is a reliable computer supervised test battery and can be repeated before and after intervention administration to assess for any resulting change. The Discrimination Reaction Time Task (measuring focused attention) and Reaction Time Task (measuring alertness needed for general cognitive task performance; Zomeren and Brouwer, 1994) were utilized in this trial from the CAB series of tests, as in previous studies these domains showed the greatest interaction with tinnitus (Wise, 2012). Focused attention requires attention to be directed toward one aspect of sensory information while excluding others, and is analogous to selective attention (Eysenck and Keane, 2015). Alertness consists of three components: (1) Expectancy, (2) Orientation to various stimuli, and (3) Readiness to produce a motor output. Decreased Reaction Time Task or Discrimination Reaction Time Task scores over time can therefore indicate loss of concentration or increased cognitive load, or inability to focus attention selectively, which can result if tinnitus is increased in magnitude.

For the Reaction Time Task, a gray square was presented in the middle of an otherwise dark/black computer screen. The visual assessment required the participant to respond as soon as possible (touching the square) when it quickly changed to a green color. The presentation lasted 200 ms and occurred after a time delay randomly varying from 1 to 4 s (1000–4000 ms).

For the Discrimination Reaction Time Task, the visual task involved watching a gray square presented in the center of a dark/black computer screen. Random visual presentations of three different colored squares occurred: red, blue or green. Participants were required to touch the square as soon as possible, registering their response, if the square changed to the target color (red) while ignoring non-target colors (blue or green). The target presentations lasted 200 ms, interspersed with 1800 ms time delays. In the auditory task condition, random auditory presentations (spoken) occurred of three different color words: Red, blue, or green. Participants responded whenever they heard the target color word (green) and ignored verbalizations of non-target color words (red or blue). Word presentation lasted ~300 ms. In the mixed visual and auditory condition, participants heard verbal instructions “The Target Is” followed by either: (1) The gray square changing in color to indicate a visual target, or (2) An auditory presentation (spoken) denoting an auditory target, color word. Whenever the target was seen or heard (depending on whether the target given was visual or auditory in nature), the participant was required to press the square as quickly as possible. While anticipating the indicated target, participants experienced randomized presentation of visual and auditory non-targets; spoken color words or visually presented color changes for the gray square. Targets were altered seven times during the assessment. The tasks resulted in assessment of pure visual reaction time (50 stimuli), pure auditory reaction time (50 stimuli), and visual and auditory reaction time (100 stimuli).

#### Assessments: qualitative interviews

At each follow-up appointment and at the end of the trial all participants were interviewed, and the interviews were digitally recorded, transcribed, and responses coded into themes (Gale et al., 2013). The interview schedule for each follow-up appointment was as follows:

1. How often did you use sounds stimuli?
2. In which particular environments did you find yourself using the sounds?
3. How is the quality of the intervention sound?
4. How you feel the sound is interacting with your tinnitus?
5. Has the quality (characteristics of your tinnitus such as the pitch, duration, fluctuation, etc.) of your tinnitus changed over the last month? If yes, how?

Additional questions asked during the final end-of-trial interview were:

6. Which of the two stimuli (BBN or nature) did you prefer the most? Why?
7. Will you be willing to wear this device as a form of tinnitus management for the next 6 months? Why or why not?
8. How can each of the sound stimuli be improved and why would this be an improvement?
9. Any other comments?

### Sample size

A power analysis indicated that 21 participants would need to enter this two-treatment crossover study. The probability was 80% that the study will detect a treatment difference at a two-sided 0.05 significance level, if the true difference between treatments was 13.0 units on the TFI. This is based on the assumption that the standard deviation of the difference in the TFI is 20.

### Randomization

The order of sound presentation for participants (Order 1 = BBN then Nature OR Order 2 = Nature then BBN) was decided using an online, free True Random Number Generator (https://www.random.org/). There were no significant differences in personality trait scores between participants placed in Order 1 compared to Order 2. Throughout the trial the same researcher tested all participants. The only blinding applied was participants were not shown the results of their tinnitus outcome measures at the different time points until the end of the trial. Blinding to intervention type could not be provided due to the distinct perceptual sound characteristics of the two sound stimuli. No tinnitus counseling was provided; participants had their hearing tests and tinnitus results explained, and instructions were provided on use of the MP3 player and how to set volume relative to their tinnitus. The nature sound trialed was that chosen by the user.

### Statistical methods

A 2 × 3 repeated-measures Analysis of Variance (ANOVA) was used to examine changes in outcome measures between the two sound types (BBN, natures sounds) at the three time points (baseline, 4 weeks of intervention and 8 weeks of intervention). All assumptions were tested for all outcomes for each independent variable to see if they were met before running ANOVA. In cases where a significant main effect was observed, Bonferroni *post-hoc* tests were administered.

For outcome measures where there was no group effect for intervention observed at 8 weeks, further bivariate correlation and ANOVA analyses of changes in outcome measures (8 weeks-baseline) was conducted in order to explore whether age, gender, and degree of hearing loss [categorized as slight, mild, moderate, moderately severe, severe, or profound (Clark, 1981) based on average of 3000, 4000, and 6000 Hz hearing thresholds bilaterally] effects were present.

In order to extract potential converging information of the different outcome measures and identify key factors influencing the effect of sound therapy administration on tinnitus over time, a Principal Component Analysis (PCA) was conducted. Changes in all outcome measures (regardless of BBN or Nature sounds) between 8 weeks and baseline as well as baseline measures of personality were included. All components with Eigenvalue >1 were extracted. Following inspection of data and the scree plot, a decision was made regarding the final number of components to be included in rotational analysis with Direct Oblimin rotation. Correlations about 0.5 were criterion used to define and load key variables to respective components and construct dimensions.

The framework method (Gale et al., 2013) was used to analyse the qualitative interviews, consisting of five steps: familiarization, identification of a thematic framework, indexing, charting, and mapping and interpretation. Familiarization involved careful listening to the digital recordings and transcribing, and re-reading the transcription. Common themes were identified in the transcripts, and in the charting phase, the data was rearranged according to theme. In the mapping and interpretative stages, the charted data was compared, and contrasted to identify patterns within the data. Quotations from participants and their thematic analysis were included in the results following standard practice in qualitative methodology (Rossman and Wilson, 1985; Onwuegbuzie and Leech, 2005).

## Results

### Participants flow and baseline data

**Figure 2:**
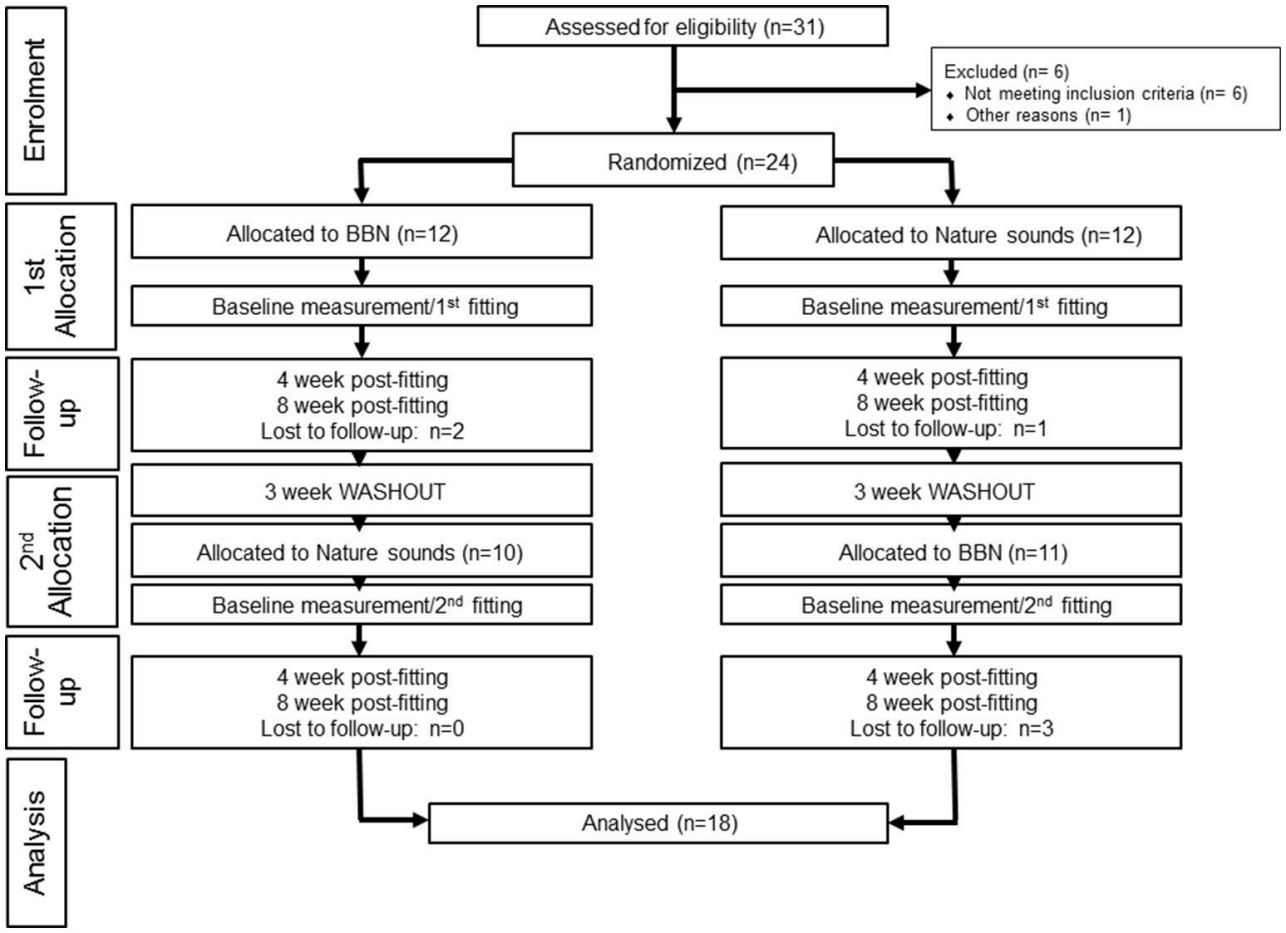
**CONSORT flow diagram of participants**.

Thirty-one participants from the University of Auckland Tinnitus Research Volunteer Database expressed interest in the trial; seven participants did not meet the criteria or were excluded for other reasons (one participant was administered the intervention sounds but the data was excluded from final analysis due to a too low baseline TFI score; removed due to basement effects). The data from 24 participants (8 females, 16 males, mean age = 56.31, range 37–65) was taken for the final trial (Figure 2). Eighteen participants (7 female, 11 male, mean age = 60.63, range 38–65) completed the trial, retention was 76%. Three participants were lost to follow-up (did not respond to emails, attend follow-up appointments and/or did not finish trialing both sounds) and three participants had early termination. Early termination of trial refers to cases where participants voluntarily expressed they wanted to stop the trial between the 4 and 8 week appointment period for one or both of the Intervention sounds, but still attended follow-up appointments.

The average baseline outcome measures of participants at the start of the trial is provided in Table 2 (individual baseline outcome measures are provided in Appendix B in Supplementary Material). The mean Tinnitus Functional Index (TFI) score of participants was 41.5 (*SD* = 15.5). All participants had experienced chronic bothersome tinnitus for a minimum of 4 years with an average length of time since tinnitus onset of 17.5 years (*SD* = 12.2, ranging from 4 to 45 years). Thirty-nine percent of participants described tinnitus quality as cricket sounds, 39% as tonal and 22% as noise. Measured tinnitus pitch ranged from 800 to 15,750 Hz, and there was no clustering observed around any particular pitch match. Fifty percent of participants had not used any form of tinnitus treatment in the past, 25% had tried one treatment and 25% had tried more than one treatment. Three out of the 18 participants (17%) wore pairs of hearing aids. When asked whether loud sounds tended to make their tinnitus worse, 42% responded that it did exacerbate it, 32% responded no and 26% did not know. Forty-two percent of participants felt that their tinnitus was reduced by music or by certain types of nature sounds (such as the noise of a waterfall, running shower water, etc.) and the remaining 58% did not know.

**Table 2:** **Average baseline characteristics of participants as measured at the start of the trial**.

		**Order 1**	**Order 2**
Demographics	Gender	Five female, four male	Two female, six male
	Age	59.3 (9.6)	56.6 (7.7)
Tinnitus characteristics	Duration (Years)	18.2 (15.5)	14 (8.5)
	Loudness rating (1–10)	6.2 (1.5)	7 (0.8)
	Annoyance rating (1–10)	5.7 (2.2)	5.6 (1.2)
	Total TFI score (weighted)	38.6 (12.5)	47.4 (17)
	LLM	3 (4)	4.8 (4.5)
	MML	6 (6)	7.4 (6.4)
Emotional/psychological	Positive emotionality	34.2 (6)	34.1 (6.7)
	Negative emotionality	16.2 (5.4)	16.5 (6.7)
	Anxiety	5 (6.4)	4.6 (5.7)
	Depression	3.1 (4)	2.9 (3.6)
	Stress	6.8 (5)	7.1 (7.7)
Personality traits	Stress reaction	7.6 (2.1)	6.3 (4.9)
	Social closeness	5.1 (2.5)	6.1 (2.8)
	Self-control	14.4 (3.1)	13.4 (3.1)
	Alienation	1.1 (1.2)	1.5 (1.5)

*Values inside brackets in columns represent one standard deviation. Order 1, BBN then Nature as order of intervention presentation for participants. Order 2, Nature then BBN as order of intervention presentation for participants*.

### Loudness and annoyance functions for sound therapy stimuli and tinnitus

**Figure 3:**
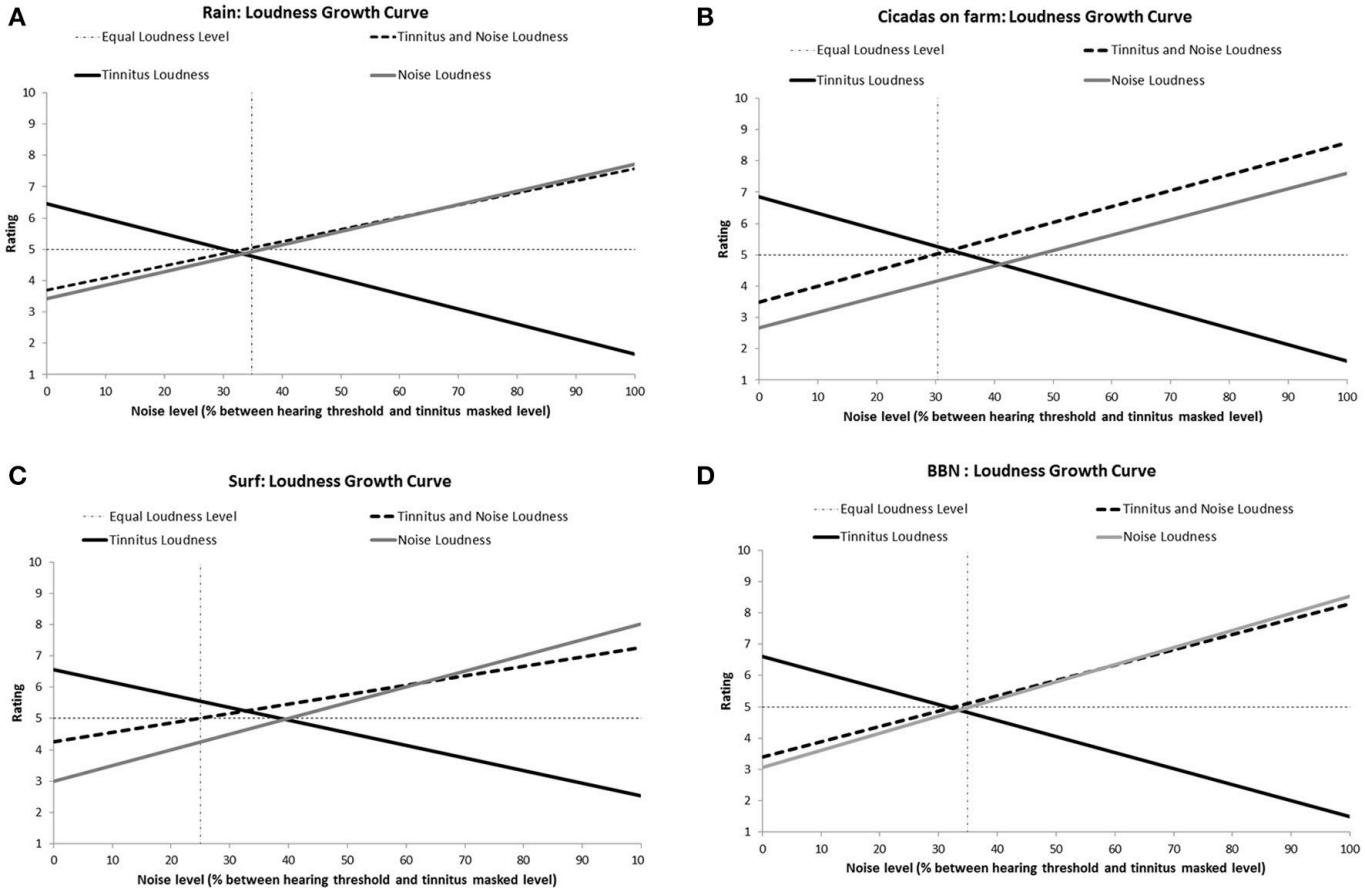
**Loudness ratings growth curves for each therapy sound [Rain (A)**, Cicadas on farm **(B)**, Surf **(C)**, BBN **(D)**] as a function of noise level (% between hearing threshold and minimum masking level for tinnitus). Loudness functions show decreases in tinnitus loudness (solid black line), increases in sound loudness (solid gray line), and increases in combined tinnitus and sound loudness as a function of sound level (dashed line). The Equal Loudness Level (vertical dashed line) defines the sound level at which both tinnitus and sound were of equal perceived loudness (tinnitus and sound loudness rating = 5).

**Figure 4:**
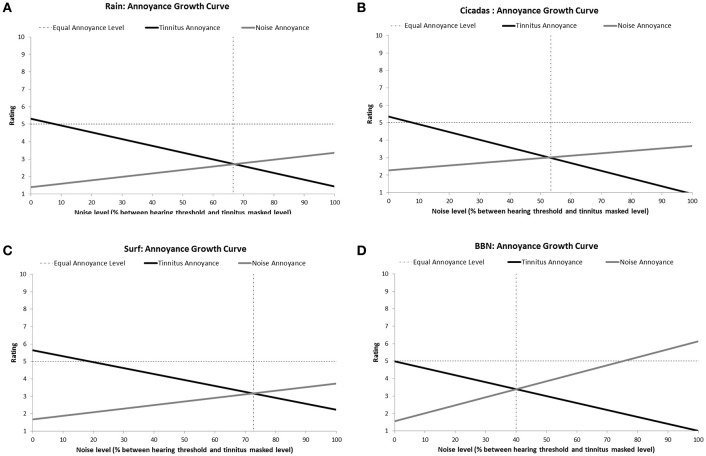
**Annoyance ratings growth curves of each therapy sound [Rain (A)**, Cicadas on Farm **(B)**, Surf **(C)**, BBN **(D)**] as a function of noise level (% between hearing threshold and minimum masking level for tinnitus]. Annoyance functions show decreases in tinnitus annoyance (solid black line) and increases in sound annoyance (solid gray line) as a function of sound level. The equal annoyance point (vertical dashed line) defines the sound level at which both tinnitus and sounds were of equal perceived annoyance (point of intersection between tinnitus annoyance and sound annoyance functions).

All the sounds resulted in decreased tinnitus loudness and annoyance, and increases in sound loudness and annoyance occurred as noise level was raised (Figures 3, 4). The average ratings for sound therapy stimuli at MML are provided in Table 3. When the sounds were ranked based on average rating changes with noise level increases, Rain was ranked #1 (equal) for tinnitus loudness decline, #4 (equal) for sound loudness growth, #1 (equal) for tinnitus annoyance decline, and #2 (equal) for sound annoyance growth. Cicadas was ranked as #1 (equal) in tinnitus loudness decline, #4 (equal) for sound loudness growth, #1 (equal) for tinnitus annoyance decline, and #1 for sound annoyance growth. Surf was ranked #4 for tinnitus loudness decline, #1 for sound loudness growth, #4 for tinnitus annoyance decline and #2 (equal) for sound annoyance growth. BBN was ranked #1 (equal) for tinnitus loudness decline, #1 for sound loudness growth, #1 (equal) for tinnitus annoyance decline, and #4 for sound annoyance growth. The Equal Loudness Level ranking was: Rain/BBN > Cicadas > Surf. The Equal Annoyance Level ranking was: Surf > Rain > Cicadas > BBN.

**Table 3:** **Ratings on a scale of 1–10 of tinnitus and sound loudness and annoyance at the MML of tinnitus**.

**Rating at MML (on scale of 1–10)**	**Rain**	**Cicadas**	**Surf**	**BBN**
Tinnitus loudness	2 (2=)	2 (2=)	3 (4)	1 (1)
Noise loudness	8 (1=)	8 (1=)	8 (1=)	9 (4)
Tinnitus annoyance	1 (1=)	1 (1=)	2 (4)	1 (1=)
Sound annoyance	3 (1)	4 (2=)	4 (2=)	6 (4)

*For example, BBN at MML was rated as being louder and more annoying than other sounds; however, the administration of BBN at this level also resulted in one of the lowest tinnitus loudness and annoyance ratings. The number in brackets represents ranking from 1 (best on measure for sound therapy) to 4 (worst on measure for sound therapy)*.

### Selection of nature sound

**Figure 5:**
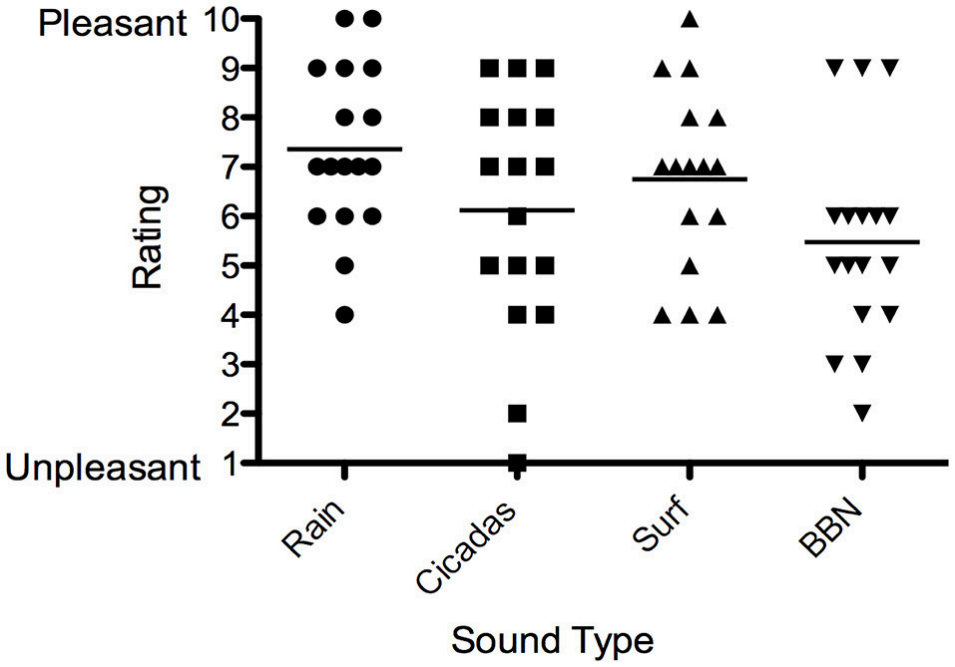
**Valence ratings of sound therapy stimuli [Rain (circles), Cicadas on Farm (squares), Surf (triangles), BBN (inverted triangles)] by participants**. Horizontal lines represent average valence ratings for each sound stimulus.

Eleven out of the 18 participants (61%) selected the Rain sound to be used in the trial, and key reasons were that it was soothing and interacted more with tinnitus. Five participants selected the Surf sound (28%) while two selected Cicadas (11%).ain had the highest valence rating (most pleasant) by participants, followed by the Surf and Cicadas respectively. BBN was the least pleasant of all the sounds (Figure 5). All participants except one (who expressed neutral feelings) reported the nature sound was pleasant, soothing, relaxing and elicited happy feelings.

### Intervention outcomes: tinnitus measures

**Figure 6:**
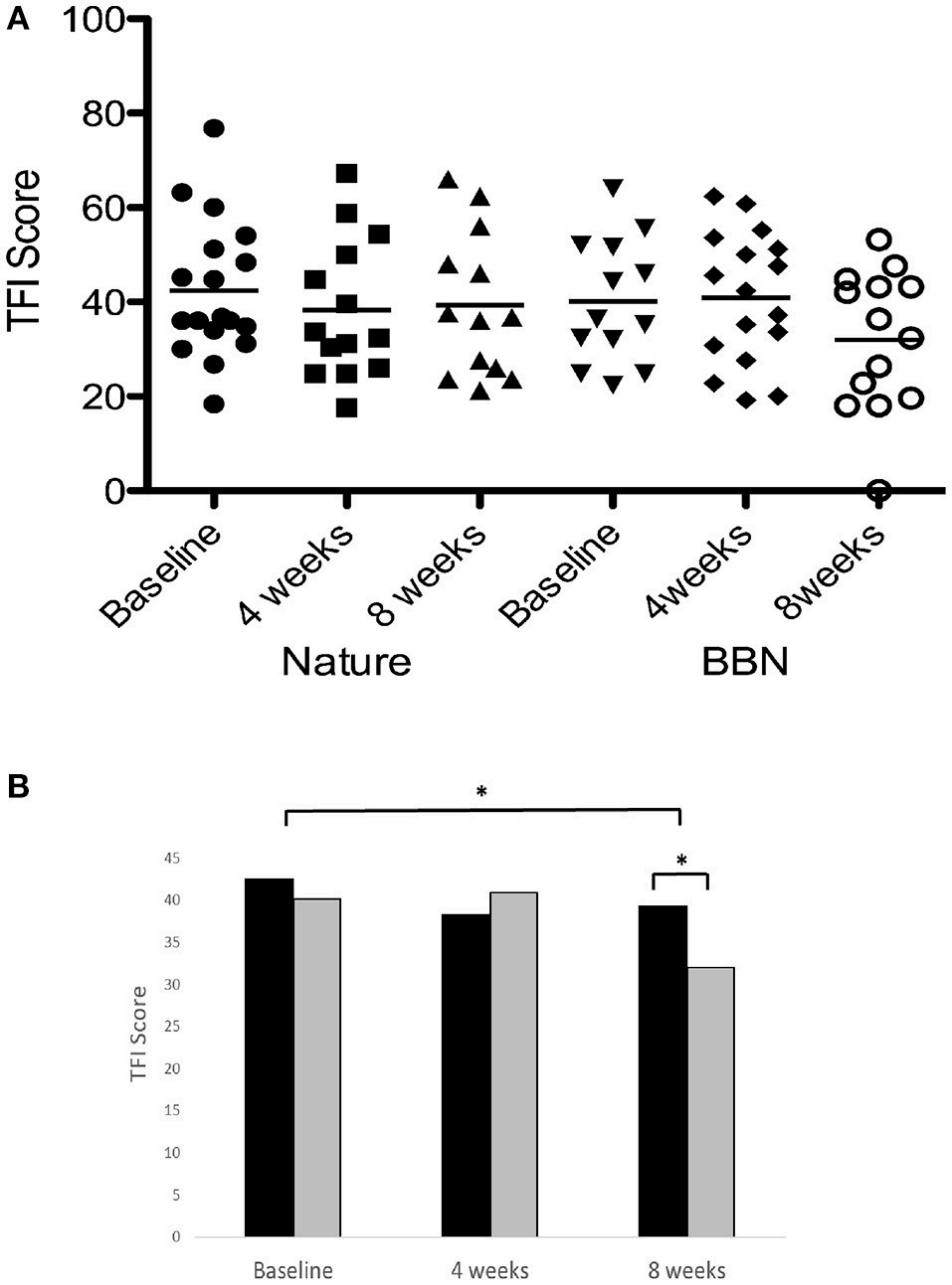
**(A)** Individual TFI scores of participants at baseline, at 4 weeks follow-up and at 8 weeks follow-up following administration of BBN and nature sound stimuli. Horizontal lines represent average TFI scores. **(B)** Average TFI scores of participants at baseline (filled circles for nature sounds; inverted triangles for BBN), at 4 weeks follow-up (squares for nature sounds; diamonds for BBN) and at 8 weeks follow-up (triangles for nature sounds; open circles for BBN) following administration of BBN (black) and nature (gray) sound stimuli. The significant difference is indicated by ^*^p < 0.05. Error bars represent ± one standard error.

There was a significant main effect of sound therapy time on TFI scores, with a 5.7 point decrease in TFI scores at 8 weeks compared to baseline [*F*_(2, 28)_ = 4.144, *p* < 0.05; Figure 6]. There was a significant effect of sound types at 8 weeks [*F*_(1, 28)_ = 6.875, *p* < 0.05], with BBN sound administration resulting in a mean 8.2 point decrease in scores, while nature sounds resulted in a 3.2 point decrease. The small change in TFI in response to the nature sounds at 4 weeks (4.2 point decrease) was not statistically significant. There were no significant difference in tinnitus measures between before the washout (8 week follow-up appointment for the first sound) and immediately after the washout (sound fitting appointment for the second sound). There was also no effect of order: the degree of change in tinnitus outcome measures was not significantly different between the first and second sound administered.

**Figure 7:**
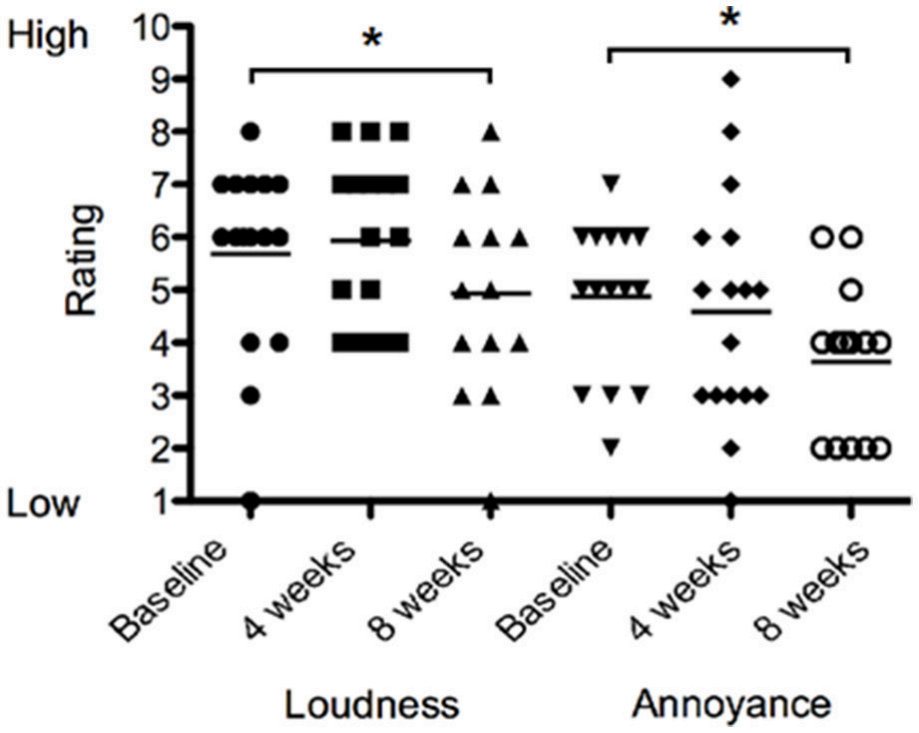
**Tinnitus loudness ratings (on a scale of 1–10, where 1 corresponded with very quiet tinnitus and 10 with extremely loud tinnitus) and annoyance ratings (on a scale of 1–10, where 1 corresponded low in distress and/or annoyance and 10 with extremely high distress) of participants at baseline (filled circles for loudness ratings; inverted triangles for annoyance ratings), at 4 weeks follow-up (squares for loudness ratings; diamonds for annoyance ratings) and at 8 weeks follow-up (triangles for loudness ratings; open circles for annoyance ratings)**. Significant differences are indicated by ^*^p < 0.05. Horizontal lines represent average rating scores.

There was no difference in loudness or annoyance ratings following sound therapy at 4 weeks compared to baseline. At 8 weeks the loudness ratings were 13% lower than at baseline irrespective of the BBN or nature sound condition [*F*_(2, 28)_ = 1.551, *p* < 0.05; Figure 7]. At 8 weeks, annoyance ratings were 25% lower than at baseline irrespective of BBN or nature sound condition [*F*_(2, 28)_ = 2.815, *p* < 0.05]. There was no significant difference in tinnitus loudness ratings and annoyance ratings between BBN or nature sound conditions at either 4 or 8 weeks.

**Figure 8:**
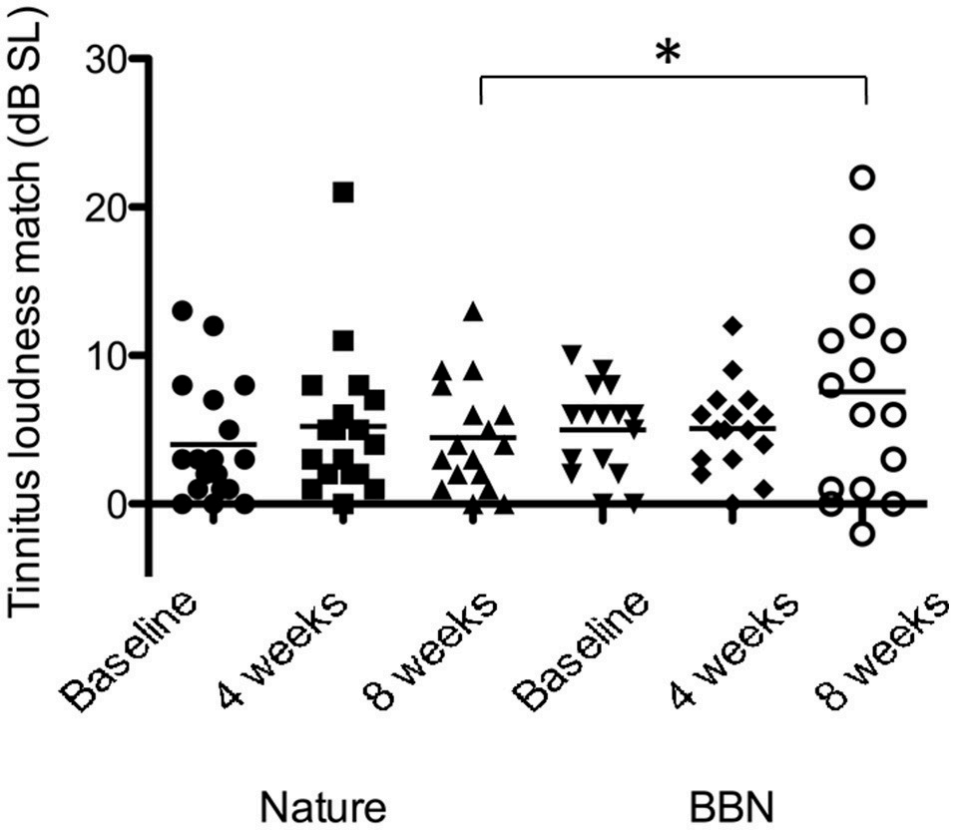
**Average LLMs (in dB SL) at baseline (filled circles for nature sounds; inverted triangles for BBN), at 4 weeks follow-up (squares for nature sounds; diamonds for BBN) and at 8 weeks follow-up (triangles for nature sounds; open circles for BBN) following administration of BBN (black) and nature (gray) sound stimuli**. Horizontal lines represent average tinnitus loudness matches (in dB SL). Significant differences are indicated by ^*^p < 0.05. Error bars represent ± one standard error.

There was a significant effect of sound type on psychoacoustic loudness level matches at 8 weeks [*F*_(1, 28)_ = 3.134, *p* < 0.05], with BBN sound administration resulting in a greater mean increase in loudness level match (2.6 dB increase in LLM) while nature sounds had slight increase (0.47 dB increase in LLM; Figure 8). There was no significant difference between BBN or nature sound conditions at 4 weeks. There was no significant main effect of sound therapy time on tinnitus minimum masking levels between baseline and 4 weeks or baseline and 8 weeks. There was no significant change in minimum masking levels between 4 weeks and baseline. There was no significant difference between minimum masking levels with sound types at either 4 or 8 weeks.

### Intervention outcomes: psychological measures

**Figure 9:**
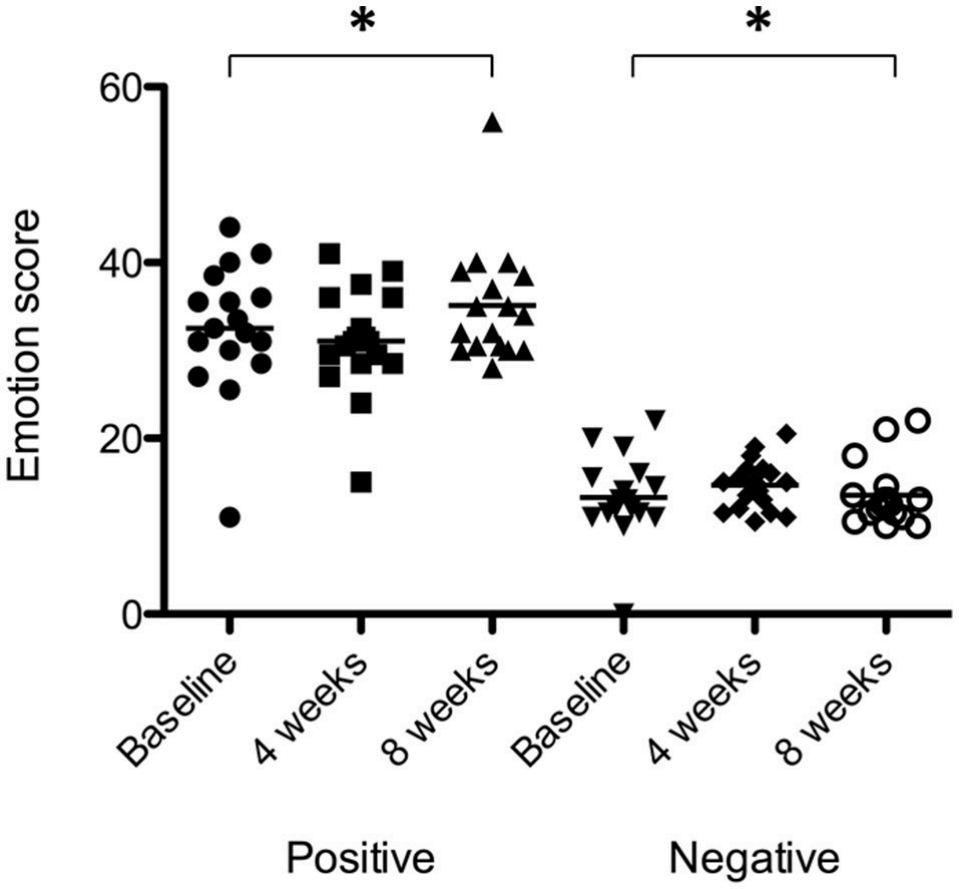
**Positive emotionality and negative emotionality scores at baseline (filled circles for positive emotionality; inverted triangles for negative emotionality), at 4 weeks follow-up (squares for positive emotionality; diamonds for negative emotionality) and at 8 weeks follow-up (triangles for positive emotionality; open circles for negative emotionality)**. Horizontal lines represent average scores. Significant differences are indicated by ^*^p < 0.05.

There was a significant effect of sound therapy time on PANAS positive emotionality scores [*F*_(2, 28)_ = 2.210, *p* < 0.05] with lower levels reported 8 weeks compared to baseline (1.4 points; Figure 9). There was no significant difference between sound types on positive emotionality scores at either 4 or 8 weeks.

There was a very small but significant effect of sound therapy time on negative emotionality scores [*F*_(2, 28)_ = 1.247, *p* < 0.05], with an increase in scores at 8 weeks compared to baseline (0.2 points). There was no change in negative emotionality scores between 4 weeks and baseline. There was no significant difference between sound types on negative emotionality scores at either 4 or 8 weeks.

**Figure 10:**
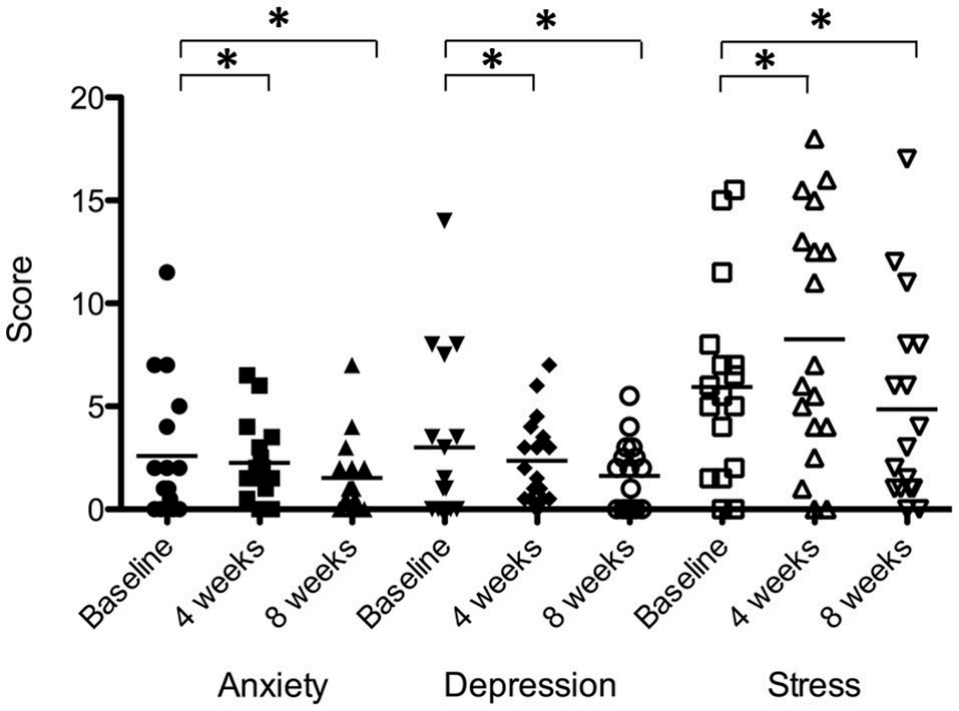
**Anxiety, depression, and stress scores of participants at baseline (filled circles for anxiety; inverted triangles for depression; open squares for stress), at 4 weeks follow-up (squares for anxiety; diamonds for depression; open triangles for stress) and at 8 weeks follow-up (triangles for anxiety; unfilled circles for depression; open inverted triangles for stress)**. Horizontal lines represent average scores. Significant differences are indicated by ^*^p < 0.05.

There was a significant effect of sound therapy time on all outcomes measures of anxiety, depression and stress (Figure 10). Reduced anxiety scores were observed between 4 weeks and baseline (0.3 points), and 8 weeks and baseline (1.1 points) [*F*_(2, 28)_ = 3.721, *p* < 0.05]; reduced depression scores were observed between 4 weeks and baseline (0.6 points), and 8 weeks and baseline (1.4 points) [*F*_(2, 28)_ = 2.44, *p* < 0.05]; stress scores were increased between 4 weeks and baseline (2.3 points), and decreased between 8 weeks and baseline (1.1 points) [*F*_(2, 28)_ = 3.01, *p* < 0.05].

There were no significant effects of sound therapy time or sound type on either attention reaction response times or attention discrimination response times.

### Principal component analysis

The Kaiser-Meyer-Olkin measure (KMO) verified the sampling adequacy for the analysis (KMO = 0.62). Bartlett's test of sphericity indicated that inter-measure correlations were sufficiently large for PCA (*p* < 0.001). The majority (87.5%) of variation in outcome variables following sound therapy administration over time were accounted for by changes in tinnitus impact on life (27%), tinnitus perceptual characteristics (9%), stress reduction/relaxation (21%), changes in positive mood (16%), and changes in negative mood (14%). This was a satisfactory amount of variation. The individual correlations/strength of loadings of each intervention outcome measure on to each principle component is provided in Table 4.

**Table 4:** **Principal components analysis (with direct oblimin rotation) loadings of intervention study outcomes**.

	**Tinnitus**		**Psychological**			***h*****^2^**
	**Component 1**	**Component 2**	**Component 3**	**Component 4**	**Component 5**	
	**Tinnitus influence—impact on life**	**Tinnitus—perceptual characteristics**	**Stress reduction/relaxation**	**Positive mood**	**Negative mood**	
TFI total score	**0.787**	**0.608**	0.076	0.027	0.003	0.995
Tinnitus loudness ratings	−0.493	**0.860**	0.075	0.005	−0.050	0.990
Tinnitus annoyance ratings	**0.984**	0.010	−0.052	−0.053	−0.085	0.982
Tinnitus loudness level match	**0.984**	0.010	−0.052	−0.053	−0.085	0.982
Tinnitus minimum masking level	0.134	**0.967**	0.047	−0.031	−0.115	0.969
Positive emotionality	−0.032	0.083	−0.310	**0.898**	0.039	0.912
Negative emotionality	0.118	0.112	−0.032	−0.318	**0.788**	0.750
Depression	−0.050	0.109	**0.601**	−**0.730**	−0.017	0.905
Anxiety	−0.171	0.053	**0.887**	−0.102	0.142	0.850
Stress	0.091	−0.037	**0.856**	−0.270	0.282	0.896
Attention reaction response times	−**0.547**	0.061	**0.584**	−0.278	−0.397	0.879
Attention discriminatory response times	−0.074	−0.447	0.258	**0.685**	−0.246	0.803
Personality: stress reaction	0.073	−0.027	−**0.730**	−0.041	**0.558**	0.853
Personality: self-control	−0.044	0.326	−0.252	**0.636**	0.481	0.808
Personality: social closeness	−0.083	−0.258	0.211	0.390	**0.773**	0.869
Personality: alienation	0.168	0.080	−0.072	−0.066	−**0.713**	0.552
% of variance explained by each factor	0.27	0.09	0.21	0.16	0.14	0.875

*Eigen-value > 1 criteria applied. Correlations above 0.5 and total variance explained by principal components are presented in bold*.

### Correlations and differences of age, gender, and hearing loss on outcome measures

The effects of age, gender, or hearing loss on outcome measure changes were investigated. Participants with mild hearing loss had a decrease in LLM (5.7 dB SL), while those with moderately severe hearing loss showed a slight increase in LLM (0.83 dB SL) between baseline and 8 weeks [*F*_(3, 15)_ = 2.32, *p* < 0.05]. During administration of BBN and natures sounds, significant differences by gender were present for negative emotionality [*F*_(1, 15)_ = 6.393, *p* < 0.05]; females displayed an increase in scores between baseline and 4 weeks (2.4 point increase), while males had a decrease in scores between baseline and 4 weeks (4.4 point decrease). Significant differences by gender were present for depression [*F*_(1, 15)_ = 3.096, *p* < 0.05], anxiety [*F*_(1, 15)_ = 5.532, *p* < 0.05], and stress [*F*_(1, 15)_ = 6.37, *p* < 0.05]. Females had a slight increase in depression scores (0.52 points) and anxiety scores (0.82 points) between baseline and 4 weeks; males had a decrease in depression scores (3.35 points) and anxiety scores (2.09 points) for the same time period. Females had an increase in stress scores (2.96 points) between baseline and 8 weeks; males had a decrease in stress scores (2.42 points) for the same time period.

### Individual differences

**Figure 11:**
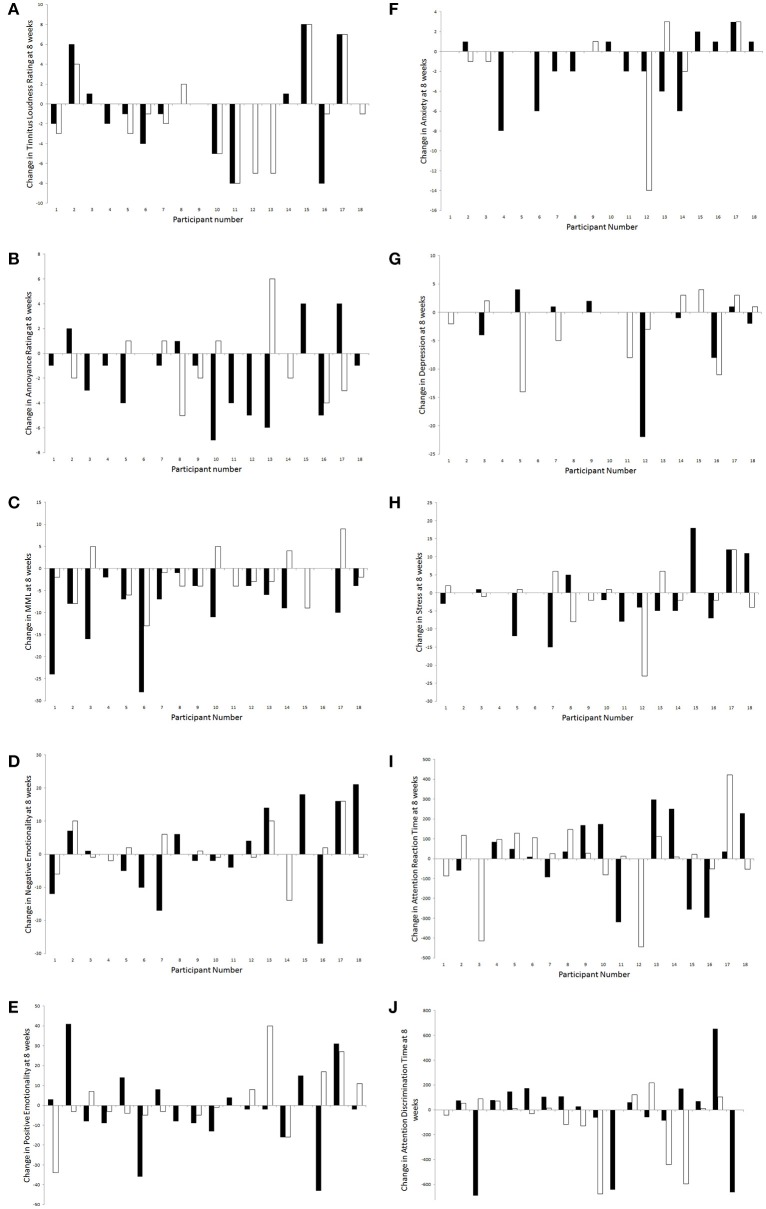
**Changes in outcome measures [(A)**, tinnitus loudness ratings; **(B)**, tinnitus annoyance ratings; **(C)**, MML; **(D)**, negative emotionality; **(E)**, positive emotionality; **(F)**, anxiety; **(G)**, depression; **(H)**, stress; **(I)**, attention reaction time; **(J)**, attention discrimination time] for each participant following administration of BBN (black) and nature (white) sound stimuli.

For all outcome measures where there were no effects of sound types after 8 weeks of trial, individual results were explored in a descriptive manner for any patterns (Figure 11). Overall, there was a considerably large amount of individual variability present in responses to sound therapy.

A greater number of participants seemed to experience a decrease in tinnitus loudness ratings at 8 weeks compared to baseline (than an increase or no change); this was slightly more likely to occur during administration of nature sound than BBN. For those who experienced an increase in tinnitus loudness rating with the presence of sound, this was most likely to occur regardless of whether BBN or nature sound was administered. Likewise more participants seemed to experience a decrease in tinnitus annoyance ratings at 8 weeks compared to baseline (than an increase or no change); however this was more likely to occur during administration of BBN sound. For those who experienced an increase in tinnitus loudness rating with the presence of sound, this was most likely to occur for a specific sound type (either BBN or nature sound, but not both).

More participants seemed to experience a decrease in MML and anxiety scores at 8 weeks compared to baseline; for both these outcome measures, a decrease was more likely to occur during administration of BBN than nature sound. For negative emotionality, positive emotionality, depression, stress, attention reaction, and discrimination response time scores, individuals were roughly equally distributed by whether there was an increase, a decrease, or no change between 8 weeks and baseline. One participant had a significant decrease in depression scores under the BBN condition; the decrease in depression scores seemed to be considerably less for nature sound administered to the same individual. Another participant had a considerable decrease in depression scores with nature sound; BBN however led to an increase in depression scores in the same person.

### Intervention outcomes: qualitative reports

Following qualitative analysis using the framework method, certain key areas emerged with regards to the sound trial. Common threads identified during the qualitative interviews are outlined below (relevant excerpts are included in Appendix C in Supplementary Material).

#### Hours and environments of use

Most participants used both sounds for the minimum amount required each day and reported usage ranged from 0.5 to 1.5 h for BBN. The nature sounds were listened to for longer periods of time: nine participants reported consistently using the nature sound for 2 h or more. One participant used the sounds at work (7 h/day). If participants were involved in engaging activities, they often let the sounds run on. The vast majority of participants used the sounds in more than one environment: 48% in quiet, usually in the evenings or in bed reading, 26% working on quiet tasks around the house, garden or in the car, and 26% at the office or doing computer work. A few participants reported experimenting with the sounds in some situations with extra sound such as TV, radio, while having conversations, or in traffic noise. The use of sounds in the presence of noise did not make the tinnitus worse.

#### Early termination of trial

For 17% of participants (three participants), the trial had to be terminated early due to significant exacerbation of tinnitus. In two out of the three cases, termination occurred during administration of BBN sound. The reason for variation was a specific life event (death of brother-in-law, disruption to sleep activity) and an incident (workplace incident exposure to loud noise). The third case terminated during nature sound administration, as they had disruption to sleep activity and did not observe any benefit.

#### Effectiveness of intervention sounds at 4 weeks

At 4 weeks 42% of participants reported experiencing a worsening of tinnitus with both sounds. Among those who reported no change in tinnitus (39%), a slightly higher proportion reported this during administration of BBN than nature sound. In contrast, slightly more participants (19%) obtained tinnitus relief with nature sound compared to BBN. Six participants felt tinnitus was exacerbated with BBN sound and nine participants with nature sound. For some participants the increase in tinnitus was not observed while the sound was playing, but immediately, or shortly, after the sound was stopped. There was no noticeable change in tinnitus for six participants listening to BBN sound and five participants listening to nature sound.

#### Effectiveness of intervention sounds at 8 weeks

At 8 weeks 52% of participants did not perceive any change. A lack of perceived change was more prevalent following administration of the nature sound than the BBN. The group that had benefit (13% of participants) was almost three times more likely to benefit from BBN than nature sounds. However, BBN was also reported more likely to make tinnitus became worse (34%). There were no statistically significant underlying differences in baseline outcome measures (e.g., baseline TFI score, LLM, etc.) or demographic factors (e.g., age, gender, etc.) between participant groups who reported benefit, no change or worse tinnitus at 8 weeks follow-up.

#### Preference of intervention sound at 8 weeks

Preference for one sound was asked, regardless of its interaction pattern with tinnitus. Of the 18 participants, only three (26%) did not have any preference. Thirty-two percent of participants preferred the BBN and a slightly higher 42% preferred the nature sound. Chi-squared tests showed that participants were not significantly more likely to choose any one of BBN, nature sound, or no preference as a response than the other. Nature sounds were reported as relaxing and had a distracting element to them that had a psychological benefit. BBN sound was described as interacting better with the tinnitus, and led to a noticeable difference in tinnitus. Among those who preferred BBN, the nature sound was commonly described as the more pleasant sound, but BBN was more efficient for treatment. In contrast, others did not like the distracting effect of the nature sound and found attention was directed toward the tinnitus instead. Participants also mentioned that they initially conceptualized BBN to be less pleasant to listen to, but discovered that it was more tolerable than they had imagined. There were no significant differences in hearing observable by sound preference; those with poorer hearing on average were less likely to have a preference although this was not statistically significant.

#### Long-term use of sound device for tinnitus management

Nine (half of total) participants were interested in continuing using the device for long-term tinnitus management and believed their tinnitus would change as a result. There was roughly equal split as to whether participants wanted to listen to BBN or nature sound over time. Two participants were interested in continuing sound therapy but did not believe their tinnitus would change. Eight participants were not interested in continuing, predominantly because there was either (1) no benefit, (2) tinnitus became louder in volume as a result of sound therapy, and/or (3) sounds made them more aware of their tinnitus as discussed previously.

#### Quality of intervention sounds

There were no concerns regarding the sound quality of both s from the majority of participants; however one participant felt their volume control increased dramatically from one step to another for the BBN.

### Relationship between intervention outcomes and qualitative reports

No trends were observed when grouping participant's tinnitus quantitative intervention outcome measures (loudness rating, annoyance rating, and LLM of tinnitus) by whether participants reported benefit or not from sound. There were also no observable trends when grouping by participant preference for an intervention sound.

## Discussion

The administration of sound therapy led to reduction in tinnitus over 8 weeks. This effect was largely due to BBN sound therapy which resulted in a 8.2 point reduction of TFI scores (Meikle et al., 2012); this was significantly different to the 3.2 point reduction following 8 weeks of Nature sound administration. The TFI reflects impact of tinnitus on quality of life (Meikle et al., 2012). Both the TFI changes were not large enough to meet one suggested clinical criterion for meaningful reduction in TFI outcome scores [a 13-point reduction (Meikle et al., 2012)] but BBN did if a different criteria of 7–8 point change (Folmer et al., 2015) was applied. For most participants sound resulted in small but significant changes in secondary outcome measures of tinnitus (reduced loudness rating scale and reduced annoyance rating scale) and psychologically related measures (increased positive emotionality, reduced anxiety, reduced depression, and reduced stress). Unlike response to rating scales, the loudness level matches increased for BBN, while there was minimal increase for loudness level matches for Nature sounds between baseline and 8 weeks follow-up. There was no significant change in MML matches following sound therapy administration. For BBN, while there was a slight decrease in loudness level matches for nature sound between baseline and 8 weeks follow-up. The results showed large individual preferences.

In this study participants played the sounds for 1–1.5 h/day, which is less than many tinnitus treatment paradigms suggest (e.g., Neuromonics Tinnitus Treatment and Tinnitus Retraining Therapy recommend 6–8 h use; Davis et al., 2008; Hanley and Davis, 2008). The time frame (8 weeks) of administration was also less than the 6 months or greater suggested by these treatments. The degree of change observed with sound may be different if used for longer periods of time per day or administered over a longer time frame (e.g., individual responses might converge or diverge over a greater amount of time).

### Individual effects (age, gender, hearing loss)

There were some interesting differences observed in gender and hearing loss with regards to some of the changes in outcome measures (Hunter and Gillen, 2009). For the psychological outcomes of negative emotionality, depression, anxiety and stress, females had an initial worsening of symptoms between baseline and 4 weeks, while males had a decrease. LLMs significantly decreased over 8 weeks among individuals with mild hearing loss (by 6 points) while those with moderately severe hearing loss actually had an increase in LLM (by slightly <1 point). The introduction of sound therapy was most beneficial in cases of mild deafferentation and/or auditory pathway damage. This may be interpreted in two ways: the tinnitus characteristics of those with lower levels of deafferentation may be more driven by attentional and psychological variables, such that new sound provides attention diversion and relief translating into lower tinnitus loudness measurements, or in instances of severe damage/deafferentation to the hearing system, sound therapy is not able to reach the appropriate cortical regions to elicit any changes, even when set a comfortable and audible listening level (Schaette et al., 2010). This has implications clinically when setting levels for sound therapy, especially when user-set. The counter to increasing levels to create greater tinnitus interaction is that if the level is set too high, there is a risk of triggering negative emotion and discomfort to the sound itself (Scott et al., 1990; Searchfield, 2014); thereby also preventing any AL shifts (Searchfield et al., 2012).

### Individual effects (personality traits)

The four personality traits examined in this study have been associated with tinnitus perception and distress. Tinnitus sufferers displaying higher levels of stress reaction, lower social closeness, lower self-control, and higher alienation than individuals with hearing loss (but not tinnitus; Rizzardo et al., 1998; Scott and Lindberg, 2000; Sirois et al., 2006; Welch and Dawes, 2008; Bartels et al., 2010; Durai et al., 2015; Durai and Searchfield, 2016). In this study, personality traits of self-control and social closeness were significantly negatively correlated, and social closeness and alienation were positively correlated. This is similar to previous findings applying the MPQ to tinnitus groups (Durai et al., 2017a). Females in this study had greater levels of social closeness than males. Males in this study had higher alienation than females. Welch and Dawes (2008) observed an elevation in alienation scores among men in their general population sample of 32-year-olds. Males also displayed higher emotional suppression scores than females in the study by Durai and Searchfield (Durai et al., 2017b). Thus, underlying personality differences appear to exist between males and females who experience tinnitus.

Participants with moderate hearing loss also had significantly higher self-control scores than those with severe hearing loss. Both genetic and environmental factors interact to create an individual's personality (Bouchard and Loehlin, 2001; Specht et al., 2011). Some of the personality traits identified in this study, such as stress reaction and social closeness, are difficult to change (Welch and Dawes, 2008). If any change is possible, it will be gradual and dependent on the age of the individual—absolute level changes have been reported to be more pronounced during adolescence and the elderly years of life, due to biological maturation, social expectations and conditioning processes (Costa and McRae, 1992; Costa and McCrae, 2006; Roberts et al., 2006; Corr and Matthews, 2009).

Personality differences can add to the heterogeneity presented in tinnitus, although this has been given little attention. It may be valuable to attempt to understand this contributory factor further by incorporating personality into assessment and for sub grouping to see how it shapes tinnitus perception, distress, and emotional response.

### Attention effects

Attention (focused attention and general alertness; Zomeren and Brouwer, 1994) was the only measurement dimension that did not change over the 8 weeks. However, there were significant correlations present between changes in reaction time task attention scores and changes in MML and stress scores at 8 weeks. At 8 weeks, changes in discrimination time task attention scores significantly correlated with changes in TFI, depression, and anxiety scores. This suggests a complex interaction between attention, cognitive, and psychological affect, tinnitus perceptual characteristics and tinnitus impact on life, as suggested in the ecological model of tinnitus (Searchfield, 2014).

### Interpretation under the adaptation level theory

**Figure 12:**
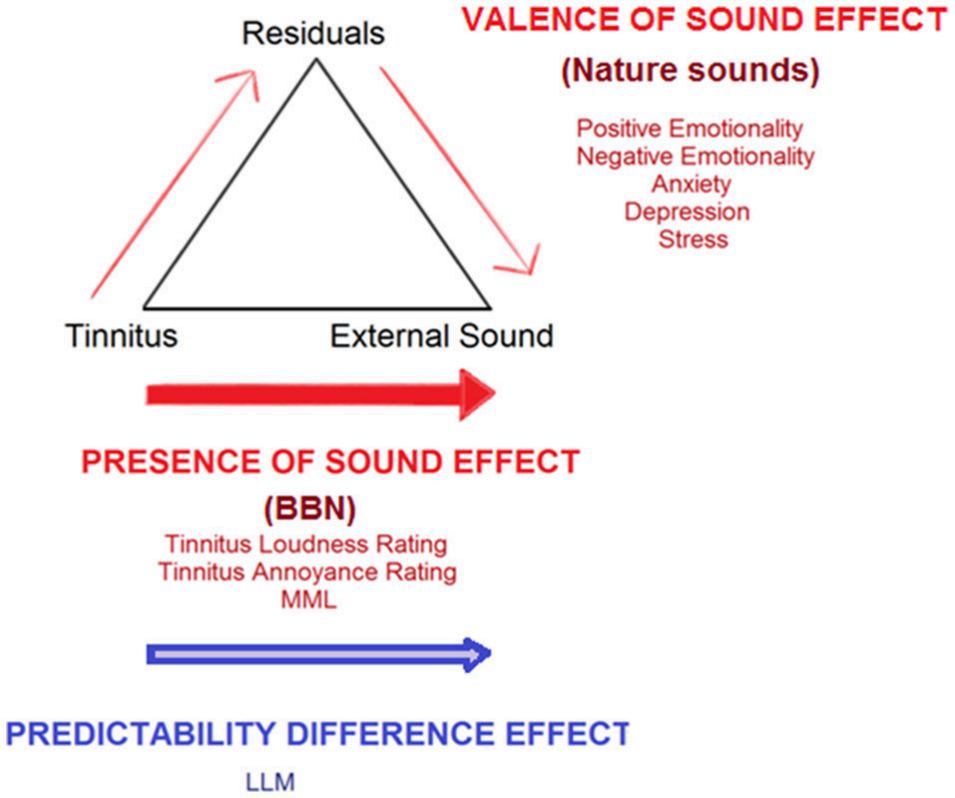
**Conceptualization of current study findings under an adaptation level theory (ALT) framework for tinnitus perception (Schecklmann et al., 2013b)**. Tinnitus is envisaged as a sensory stimulus with an existing internal adaptation level (AL) which acts as a reference point for all tinnitus-related judgments and is able to be manipulated by context and time. A high tinnitus AL results in tinnitus that is judged by the sufferer as being of high magnitude and/or eliciting high distress. Three key components set the final AL: (1) the focal component/stimuli being attended to (tinnitus), (2) background stimuli, as well as (3) residuals (various psychological and cognitive individual influences, including emotion, personality, past experiences, arousal, and level of prediction elicited by sound stimuli). The “presence of sound effect” (red arrows) illustrates steady shifts in AL away from the tinnitus and toward sound therapy stimuli, which can occur by directly increasing the weighting placed on external sound, via attention, and auditory streaming shifts. A valence of sound effect increases weighting placed on external sound via the residual pathway. The latter occurs as external sounds provide psychological relief from tinnitus and can counteract tinnitus-related negative emotions, anxiety, stress, and depression, thereby creating a facilitating residual effect which reduces tinnitus severity. The “predictability difference effect” (blue arrow) illustrates a potential difference between BBN and nature sounds in terms of the amount of prediction errors elicited; this may also influence the degree of adaptation each sound undergoes over time. Shifts in AL away from tinnitus toward external sound would discontinue upon adaptation of the auditory system to the external sound itself. Auditory system adaptation to BBN and natural sound therapy may occur at different rates. Adaptation to BBN occurs sometime between 4 and 8 weeks after the first introduction of the sound, leading to the need to increase the sound level required to match tinnitus in Loudness Level Matching. There may be small but immediate valence effects, but nature sounds due to their intermittent nature may take longer to reach peak adaptation, such that at 8 weeks no change in Loudness Level Matching may be observed.

Under the ALT model, the “presence of sound effect” (decrease in tinnitus outcomes after administration of either sound therapy stimuli) suggests a shifting of internal AL steadily away from the tinnitus and towards background noise stimulus (Figure 12). This may occur due to component weighting shifts and attention diversion. Increased positive psychological benefits may also create a facilitating residual effect, which also shifts AL. It is possible that characteristics of specific sound stimuli may work by placing greater emphasis on altering one pathway than another (e.g., BBN has been reported to aid in attention diversion; nature sounds were reported as eliciting high valence emotions). Durai et al. (2015) explored the possibility that tinnitus distress and loudness may be underlined by different perceptual and decision making processes that can be represented by two distinct adaptation levels. An AL can exist for any sensory modality, but also within each modality (Helson, 1948, 1964; Coren and Ward, 1989; Masuyama, 1994). The AL for distress might be more prone to contextual and indirect psychological influences, given the complexity of non-auditory region involvement such as the emotional, arousal, attention, and memory networks (Zenner et al., 2006; Jastreboff, 2007; De Ridder et al., 2011; Kaltenbach, 2011). De Ridder et al. (2014) have outlined a “tinnitus core” sub-network within the brain. It has been suggested that the minimal set of brain areas that needs to be simultaneously active in order for tinnitus to be consciously perceived. Affective components of tinnitus are represented by additional and overlapping networks. There is a possibility that tinnitus signal AL weighting decreases via the direct pathway toward external sound (involvement of core networks) while the affective component decreases occur via a residual pathway.

It is postulated that high levels of stress reaction, low social closeness, low self-control and high alienation may act as “maladaptive” personality residuals under this framework, diverting attention and auditory processing resources toward the tinnitus, thus increasing its AL weighting. The subsequent co-activation of various sub networks encoding tinnitus characteristics in the cortex with increased awareness/salience might then potentially explain the relationship between personality trait and psychoacoustic tinnitus characteristics (De Ridder et al., 2014, 2015). The relationship of attention within ALT is complex and it is difficult to determine weighting changes directly, due to the overlapping nature of networks possibly involved.

ALT stresses the active interaction between an individual experiencing tinnitus, cognition and their environment (both the immediate surroundings and broader factors, including their culture, beliefs, work, and social environment). The influence of the environment and health factors was evident in qualitative reports by participants, e.g., “Still feeling sick from flu, not feeling well at all, so not sure how accurate tinnitus perception might be,” “Stressed at work, because in my view I feel tinnitus is stress or noise related, so hear it more.” The physical location as well as time of day can alter the magnitude of tinnitus. Overall the success of sound appears to be partially related to individual influences, which interact to determine final tinnitus magnitude and its impact. It is not yet possible clinically to prescribe sounds that are tailored to an individual's tinnitus with confidence that they are the best sounds. However, the use of the rating functions described in this study may assist selection of sound type for therapy.

### Factors influencing sound therapy effect on tinnitus

Two components relating to sound therapy effects on tinnitus were interpreted, influencing impact of life such as presence, distress, and reactions to tinnitus (encompassing TFI scores, annoyance ratings, and LLMs) in addition to altering perceptual tinnitus characteristics such as subjective loudness and maskability (TFI scores, loudness ratings, and MML). However, we also cannot rule out placebo effects on either the qualitative or quantitative results, in scenarios where placebo effects are relevant, choice over treatment can increase these effects (Geers and Rose, 2011) which may account for qualitative preferences for the nature sounds, but does not account for the greater effects on the TFI with the BBN sound. TFI scores were the only variable which loaded onto both components of impact of life and perceptual characteristics. This is in line with one of the aims of the TFI, which is to comprehensively cover the broad range of symptoms associated with tinnitus severity (Meikle et al., 2012).

Based on the pattern of results it was also reasoned that three residuals of sound therapy effects on tinnitus were stress reduction/relaxation, and positive mood and negative mood. Under broad classification, the components map well onto the ALT model explanation of tinnitus-related and residual psychology-related effects of sound therapy discussed. The discrepancy in mood change (both positive and negative) in relation to sound therapy administration is interesting. Negative Emotionality and the personality trait of Social Closeness loaded strongly positively on the negative mood dimension, while Alienation as a personality trait loaded strongly negatively. In contrast, strong negative loadings of Stress Reaction on Stress reduction/relaxation and Self Control moderately positively loaded on positive mood. One possible interpretation is that stress and self-control are indices for discerning subgroups of individuals with exacerbated tinnitus following sound therapy. The sequence of events resulting in increased tinnitus may follow the indirect residual pathway (driven by an increase in negative affect) and the presence of certain underlying personality trait levels (e.g., social closeness, stress reaction) may determine the extent to which this pathway occurs and the magnitude of shifts in weighting toward tinnitus. However, this is only speculation and further research is needed in this regard.

Attention reaction response times loaded moderately negatively on tinnitus impact of life and moderately positively on stress reduction/relaxation. Attention discriminatory response times loaded moderately positively on positive mood. One possible explanation for this observation is that decreased tinnitus impact and increased psychological well-being in general may be related to increased attentional response times. Various studies suggest that reaction time is shorter under conditions of physiological stress (Desiderato, 1964; Ohmura et al., 2009).

### Sound adaptation as a confound

The loudness level match is commonly used to psychoacoustically measure changes in tinnitus; however its interpretation in cases where external sound is administered for long periods of time can be difficult. Discrepancies between subjective loudness rating scores and loudness level match measures have been observed in the past, and termed the tinnitus loudness paradox (Penner, 1986; Henry and Meikle, 1996; Searchfield et al., 2012). Interpreted under ALT, the loudness paradox arises because subjective loudness judgments estimate the current tinnitus AL: it is made in a sound proof booth with no contextual noise stimuli (Penner, 1986; Henry and Meikle, 1996). In contrast, the objective match is made when an external test stimulus is introduced and the individual has to match it with the existing tinnitus AL. If the AL is initially set high, the matching sound level does not have to be increased as much before it is perceived as being of equal loudness as the tinnitus. However, if the matching sound undergoes adaptation to sound over time it would appear quieter, and would therefore have to be raised in order to match the intensity of tinnitus loudness (which undergoes slower adaptation; Searchfield et al., 2012; Durai et al., 2015). The auditory system may adjust to sound therapy stimuli over time; this would eventually stop further AL shifts and/or result in shifts back toward tinnitus.

It is highly likely that adaptation to the intervention sound may confound the interpretation of loudness level matches in this study. Underlying neural changes can occur through gain control, or adjustment of input-output sound functions of auditory neurons (Marks and Arieh, 2006; Robinson and McAlpine, 2009). Studies have observed stimulus-specific adaptation effects at all levels of the auditory system from early auditory encoding (Marks and Arieh, 2006) to the auditory cortex (Robinson and McAlpine, 2009; Rabinowitz et al., 2011). Adaptation of the auditory system to BBN and nature sounds may occur at different rates. Because BBN is a predictable sound, it may be adapted to at a faster rate, and lead to an increase when an external sound is used to match tinnitus in loudness level matching. Unpredictable natural sounds are adapted to more slowly; therefore no change in loudness level match is obtained.

It is possible that intermittent tinnitus masking may not appear to alter tinnitus due to auditory continuity effects (Näätänen et al., 2001) whereby the brain “fills in the gap' where masking sound is applied and tinnitus appears as a continuous percept. This learning effect involves several networks in the brain that overlap with that of tinnitus, including the limbic structures, basal ganglia, and prefrontal cortex (Hassler, 1978). Davis et al. (2007) observed more consistent benefit over 12 months if Neuromonics Tinnitus Treatment involved masking of tinnitus for the first 2 months followed by intermittent perception of tinnitus compared to where there is intermittent perception of tinnitus throughout treatment. It may be useful to run future trials in which the temporal structure of sounds are changed often to maintain novelty and prevent sound adaptation, continuity illusion, and facilitate AL shifts toward external sound (Schreitmüller et al., 2013).

### Clinical implications and sub grouping of tinnitus characteristics

Participants often demonstrated a clear preference for one nature sound over the others in the initial selection task; mostly Rain (highest valence ratings by participants and reported as having greater interaction with tinnitus, possibly due to its broad frequency spectrum, and more consistent nature) over Surf or Cicadas. Similar loudness and annoyance growth curves for tinnitus and interactions between all sounds (nature and BBN) were present with increasing volume levels. However, BBN had higher sound annoyance rating at tinnitus masking level than all three nature sounds. BBN sound therapy has been recommended (Jastreboff and Hazell, 2004) as it is proposed to be more easily tolerated, neutral in nature, and better for facilitating habituation than tones or NBN (Jastreboff, 1999; Jastreboff and Hazell, 2004). The results of this study would agree with these suggestions, although we believe the process of tinnitus adaptation is more complex than habituation (Searchfield et al., 2012). Also, considering the BBN had a stronger effect on adaptation level while nature sounds influence residual emotion pathways, and nature sounds are more accepted (Schreitmüller et al., 2013), another clinical application would be the combing the two sounds or staging their use.

Sixteen percent of the participants experienced an exacerbation of tinnitus sufficient enough to terminate the trial early; however, this was mainly due to external situational factors or incidents. It was not possible to identify any characteristics (e.g., personality trait, age, gender, duration of tinnitus, other tinnitus variables) which isolated these individuals from others in the study. From the qualitative reports at 4 weeks after administration, there were subgroups in self-reported response to sound therapy: 42% had worsening of tinnitus, 39% no change, and 19% obtained relief. Moreover, at 8 weeks after administration, there was variation in responses: 52% had worsening, 34% had no change, and 13% had relief from sounds. The number of participants reporting benefit was lower than anticipated based on hearing aid (Folmer and Carroll, 2006; Hobson et al., 2010; Searchfield et al., 2010) and tinnitus aid (Bauer and Brozoski, 2011; Barozzi et al., 2016) studies. This may, at least in part, be due to mode of sound delivery. MP3 players and earbuds were used as a lower cost intervention than tinnitus aids. Improvements in implementation of MP3 players from a previous study were made based on participant reports (Durai et al., unpublished manuscript); an easier user interface was implemented by switching from Apple iPod shuffles to the Philips ViBE MP3 Player (with more accessible manual controls) and use of retaining hooks with the earbuds. However, even these improvements resulted in less use than hearing aids. Patient reports suggest the MP3 players were used 1–1.5 h per day, significantly less than that usually recommended for sound therapy using ear-level devices (6–8 h per day; Jastreboff, 1999; Jastreboff and Jastreboff, 2000). The sounds also did not compensate for hearing loss. Threshold adjusted noise (Searchfield et al., 2002) is implemented in several tinnitus aids (e.g., Siemens, Phonak, Oticon). The flat frequency response we used may have led to less interaction with tinnitus in the region of hearing loss, but would be similar for both the intervention sounds. Tinnitus aids can use sound in a number of ways through inbuilt sounds or by streaming sounds (Searchfield, 2016). A future trial should build on the findings in sound selection described here using tinnitus aids streaming sounds that are downloaded to tablet computers or smartphones, e.g., from hearing aid manufacturers Apps (e.g., Tinnitus Balance App, https://www.phonak.com/us/en/support/apps.html) or independent online sources (e.g., TinnitusTunes, http://www.tinnitustunes.com). Another factor to consider is the long duration of tinnitus (mean time since onset was 17 years) among participants in this study. Tinnitus neural networks tend to change as tinnitus duration increases (Schlee et al., 2009; Vanneste et al., 2011) which may impose limitations on brain plasticity and the extent of change triggered by the presence of sound.

Interestingly, there was no correlation between the reported effects of sound or preference of sound and the intervention outcomes measured in this clinical trial. These findings highlight that qualitative and quantitative measures do not always clinically equate. Melin et al. (1987) observed similar discrepancies in their study in which hearing aids were fit to individuals with tinnitus for 6 weeks. The results were explained in terms of demand characteristics: when asked during interviews, subjects may tend to exaggerate (or underestimate) the ability of the intervention to change tinnitus, while the scaling may not be as sensitive to these effects. The extent of this reporting bias might differ between numerical rating scales and qualitative interviews. The inclusion of systematic qualitative methods in sound therapy treatment paradigms may be more advantageous than quantitative measures in identifying shifts in environmental factors or significant change in factors outside of the individual which can influence tinnitus (Malterud, 2001), such as individual health or stress determinants (e.g., changes in tinnitus characteristics present as a result of illness, as reported in this trial) or to identify concerns which arise (e.g., situational factors which may result in discontinuation of sound, as reported in this trial).

Subgroups have been identified among tinnitus sufferers, which vary based on pathophysiology, perceptual features, co-morbid conditions, and how they respond to specific treatments (Stouffer and Tyler, 1990; Lockwood et al., 2002; Heller, 2003; Tyler et al., 2008). The results support the existence of different classes of tinnitus, and the ALT may eventually be most supported as a framework for only a particular tinnitus subgroup. Both auditory and non-auditory residual factors may shift the AL more readily among such individuals. Moreover, age, gender, hearing loss, personality traits, and duration of sound therapy may all (hypothetically) be factors which can help delineate individuals.

The measures used in this study were selected based on validity and usefulness from past pilot studies. It is acknowledged that there are other standardized measures available such as the Clinical Global Impression Scale (Busner and Targum, 2007) and the COSIT (Dillon et al., 1997; Searchfield, 2016) for measuring perceived improvement of tinnitus. In this trial tinnitus counseling was deliberately not provided. Current tinnitus treatment paradigms such as Tinnitus Activities Treatment (Tyler et al., 2007) and Tinnitus Retraining Therapy (Jastreboff and Jastreboff, 2000; Jastreboff and Hazell, 2004; Jastreboff, 2007) use sound therapy alongside counseling of some form. Some trials of these therapies have been criticized (Sandlin and Olsson, 1999; McKenna and Irwin, 2008) as the benefits of sound therapy, over and above counseling, have not been determined. The results reported here are independent of counseling but it is strongly advocated that the use of sound clinically should be in addition to (not instead of) counseling. Sound therapy involves more than passive exposure to sound (Bauer and Brozoski, 2008; Davis et al., 2008), and participants need to be informed of this, and counseled also about how to use the sound. For some of the participants sound therapy ended up diverting attention toward the tinnitus instead. Individually tailoring sound therapy and counseling to target different components of the ALT would be expected to demonstrate an additive effect in shifting AL weighting away from tinnitus if administered together, than if each was administered in isolation. Attention training (Searchfield et al., 2007; Wise, 2012; Wise et al., 2016) might enhance adaptation to tinnitus through the presence of sound effect, while psychoeducation (or mindfulness or Cognitive Behavioral Therapy; Sweetow, 1984, 1986; Martinez-Devesa et al., 2010) might increase tinnitus adaptation through the valence sound effect.

## Conclusions

Overall, the presence of sound had a positive effect on the TFI; after 8 weeks of administration, sound therapy with BBN resulted in a greater reduction of TFI than nature sound. The positive effect of sound on tinnitus was supported by secondary tinnitus and psychological-related outcome measures, but not interviews. BBN and nature sounds did not differ significantly on secondary outcome measures of tinnitus, emotion, attention, and psychological state after 8 weeks of administration. Interpreted under an ALT framework, internal AL weighting shifts away from the tinnitus signal and toward the sound therapy stimuli may occur, via a direct pathway toward external sound (involvement of core networks) while the affective component of tinnitus decreases via the residual pathway. The auditory system may adjust to sound therapy stimuli over time; this may eventually stop further AL shifts and result in shifts back toward tinnitus. It such cases predictable BBN might undergo loudness adaptation at a faster rate than unpredictable nature sounds.

This study provides further evidence for the heterogeneous nature of tinnitus. ALT appears to provide a framework for sound selection that could be applied to improve future sound-therapies. In this study, the selection of sound therapy stimuli by individuals was found to be, at least in part, governed by certain characteristics of the stimuli itself. Within a clinical setting, it is important to understand individual variation and that each person presents with different needs. Individual preferences were shown within this study that might be applied to improve outcomes if known apriori. It may be beneficial to have a wide range of sounds available in the clinic. The results of the Principal Component Analysis and ALT model interpretation are both compatible with an ecological framework of tinnitus, a multitude of factors (e.g., attention and personality, characteristics of and preference for sound stimuli) appeared to determine the magnitude and experience of tinnitus at any one time. Regular qualitative assessments will allow for a more comprehensive picture to be obtained regarding various factors influencing sound success. Selecting sounds based on the ALT model would involve weighing treatment sound stimuli and sound levels based on sound valence ratings, tinnitus and sound loudness and annoyance (dependent on the individual's profile at a particular point in time) as well as alternating presentation of sounds that evoke positive feelings (through the valence sound effect) and sounds with high interaction with tinnitus (for a presence of sound effect) over time, dependent on the individual's profile at a particular point in time. Trials of sound therapy selection based on Adaptation Level Therapy are needed.

## Ethics statement

Fifty individuals were invited by email invitations sent out to randomly selected members on the University of Auckland Tinnitus Research Volunteer Database (372 people from throughout New Zealand, majority from within Auckland). These are individuals with debilitating tinnitus who are interested in volunteering for tinnitus studies and clinical trials related to tinnitus relief. The inclusion criteria were: adults aged between 18 and 69 years residing in the Auckland region (NZ), constant tinnitus, and a minimum score of 38 on Tinnitus Functional Index (TFI), normal middle ear function, and a maximum of a moderate degree of hearing loss (<70 dB loss on average across frequencies). All subjects gave written informed consent in accordance with the Declaration of Helsinki. Participants read the Participant Information Sheet which listed any risks, benefits and layout of the trial as well as contact details. If participants were interested in continuing, a consent form was signed at the start of the trial.

## Author contributions

MD was involved in manuscript preparation, collection of environmental sound recordings, development of sound therapy stimuli, sound therapy administration, data collection, and analysis. GS was involved in manuscript preparation, collection of environmental sound recordings, advise in development of sound therapy, and study design.

## Funding

The author(s) disclosed receipt of the following financial support for the research, authorship, and/or publication of this article: Deafness Research Foundation New Zealand.

### Conflict of interest statement

GS is the scientific director of the University of Auckland Hearing and Tinnitus Clinic and TinnitusTunes, an online Tinnitus Therapy resource. The other author declare that the research was conducted in the absence of any commercial or financial relationships that could be construed as a potential conflict of interest.

## Abbreviations

ALT: Adaptation Level Theory
ANOVA: Analysis of Variance
ANZCTR: Australian New Zealand Clinical Trial Registry
BBN: Broadband Noise
CAB: Comprehensive Attention Battery
DASS, Depression: Anxiety and Stress Scale
EAP: Equal Annoyance Point
ELP: Equal Loudness Point
Hz: Hertz
LLM: Loudness Level Match
MML: Minimum Masking Level
MPQ: Multidimensional Personality Questionnaire
NZ: New Zealand
PANAS: Positive and Negative Affect Scale
TCHQ: Tinnitus Case History Questionnaire
TFI: Tinnitus Functional Index
THQ: Tinnitus Handicap Questionnaire
COSIT: Client Oriented Scale of Improvement for Tinnitus.
