# Corrigendum: A Mixed-Methods Trial of Broad Band Noise and Nature Sounds for Tinnitus Therapy: Group and Individual Responses Modeled under the Adaptation Level Theory of Tinnitus

**DOI:** 10.3389/fnagi.2017.00116

**Published:** 2017-05-09

**Authors:** Mithila Durai, Grant D. Searchfield

**Affiliations:** ^1^Section of Audiology, Eisdell Moore Centre, University of AucklandAuckland, New Zealand; ^2^Center for Brain Research, University of AucklandAuckland, New Zealand; ^3^Brain Research New ZealandAuckland, New Zealand

**Keywords:** clinical trial, tinnitus, auditory perception, adaptation, psychoacoustics, ecology model, sound therapy

**Figure/Table Legend**

In the original article, there was a mistake in the legend **Figure 12** as published. The reference for the figure should be Searchfield et al. (2012) instead of Schecklmann et al. (2013b). The correct legend appears below.

**FIGURE 12 | Conceptualization of current study findings under an adaptation level theory (ALT) framework for tinnitus perception (Searchfield et al., 2012)**. Tinnitus is envisaged as a sensory stimulus with an existing internal adaptation level (AL) which acts as a reference point for all tinnitus-related judgments and is able to be manipulated by context and time. A high tinnitus AL results in tinnitus that is judged by the sufferer as being of high magnitude and/or eliciting high distress. Three key components set the final AL: (1) the focal component/stimuli being attended to (tinnitus), (2) background stimuli, as well as (3) residuals (various psychological and cognitive individual influences, including emotion, personality, past experiences, arousal, and level of prediction elicited by sound stimuli). The “presence of sound effect” (red arrows) illustrates steady shifts in AL away from the tinnitus and toward sound therapy stimuli, which can occur by directly increasing the weighting placed on external sound, via attention, and auditory streaming shifts. A valence of sound effect increases weighting placed on external sound via the residual pathway. The latter occurs as external sounds provide psychological relief from tinnitus and can counteract tinnitus-related negative emotions, anxiety, stress, and depression, thereby creating a facilitating residual effect which reduces tinnitus severity. The “predictability difference effect” (blue arrow) illustrates a potential difference between BBN and nature sounds in terms of the amount of prediction errors elicited; this may also influence the degree of adaptation each sound undergoes over time. Shifts in AL away from tinnitus toward external sound would discontinue upon adaptation of the auditory system to the external sound itself. Auditory system adaptation to BBN and natural sound therapy may occur at different rates. Adaptation to BBN occurs sometime between 4 and 8 weeks after the first introduction of the sound, leading to the need to increase the sound level required to match tinnitus in Loudness Level Matching. There may be small but immediate valence effects, but nature sounds due to their intermittent nature may take longer to reach peak adaptation, such that at 8 weeks no change in Loudness Level Matching may be observed.

**Incorrect Citation**

In the original article, the citation for the text in the final paragraph under Discussion, Sound Adaptation as a Confound, “Changed often to maintain novelty and prevent sound adaptation, continuity illusion, and facilitate AL shifts toward external sound” was incorrectly cited as (Schreitmüller et al., 2013). It should be (Searchfield et al., 2012).

**Text Correction**

In the original article there was an error. The paragraph was “There were no concerns regarding the sound quality of both s from the majority of participants; however one participant felt their volume control increased dramatically from one step to another for the BBN.” A correction has been made to Results, Intervention Outcomes: Qualitative Reports, Quality of intervention sounds: “There were no concerns regarding the sound quality of both sounds from the majority of participants; however one participant felt their volume control increased dramatically from one step to another for the BBN.”

The authors apologize for these errors and state that this does not change the scientific conclusions of the article in any way.

## Conflict of interest statement

The authors declare that the research was conducted in the absence of any commercial or financial relationships that could be construed as a potential conflict of interest.
