# Supplementary material for: A Mixed-Methods Trial of Broad Band Noise and Nature Sounds for Tinnitus Therapy: Group and Individual Responses Modeled under the Adaptation Level Theory of Tinnitus
Source: Front Aging Neurosci. 2017 Mar 9;9:44. doi: 10.3389/fnagi.2017.00044 (PMC5343046; doi:10.3389/fnagi.2017.00044)
Supplement: Supplementary file 1 [file DataSheet1.DOCX]

**Appendix A. Intervention sound stimuli parameters**

1. **Rain**

A

**
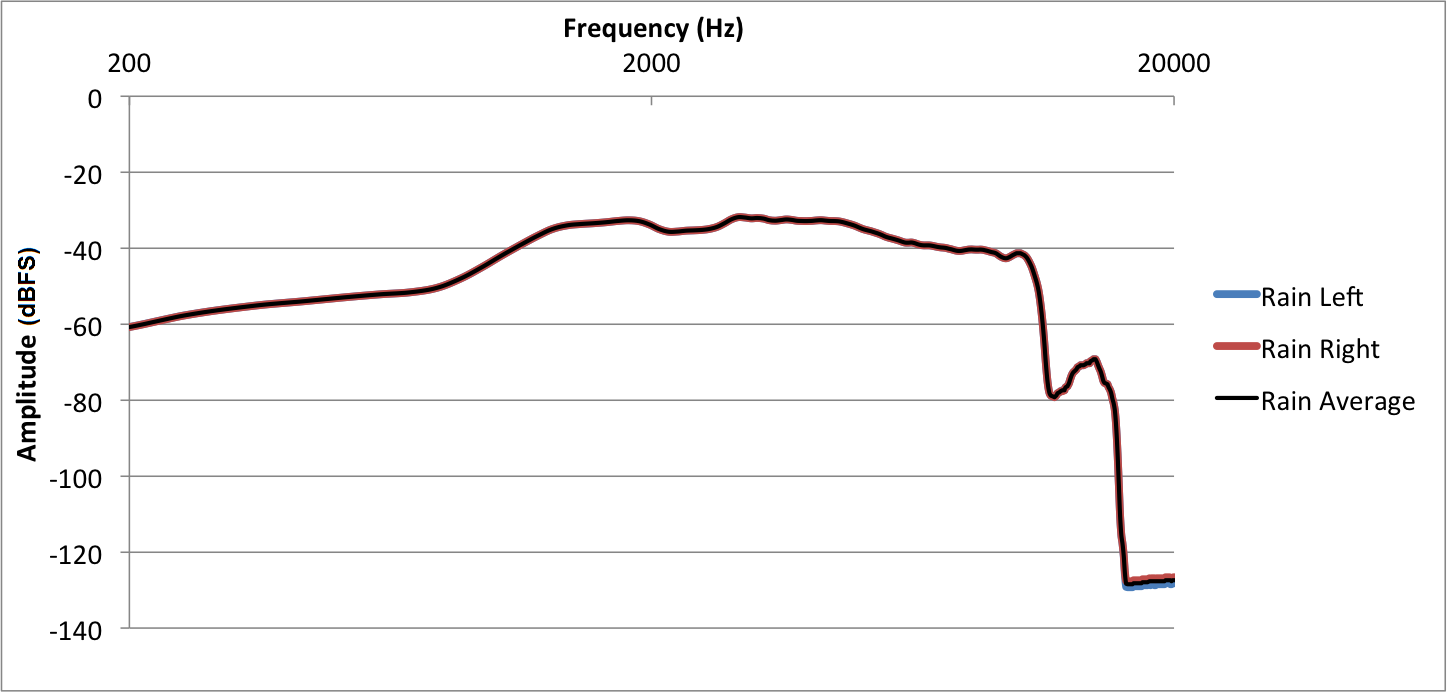
**

B

**
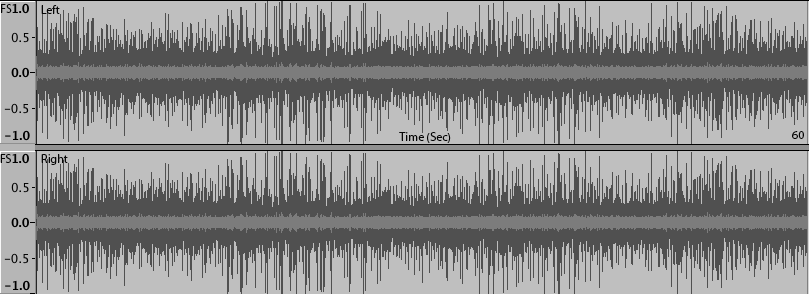
**

C

**
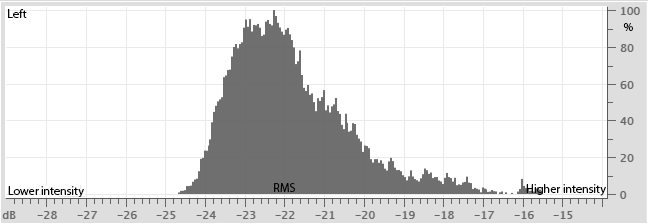
**

D


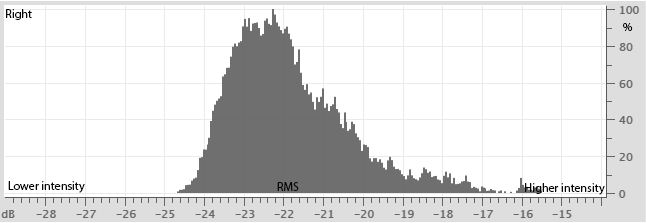


Acoustic characteristics of the intervention sound “Rain”. (A) Amplitude spectra (dB FS, decibels Full Scale) for each ear and average between ears. The spectrum recorded as a MP3 file gently rises up to 2 kHz, is roughly flat between 2 and 14 kHz, rolling off at 14 kHz and 16 kHz (16 kHz as per MP3 psychoacoustic function). (B) Amplitude (FS, Full Scale ratio) over time (60 seconds) for each ear. The sound fluctuates with time. (C) Prevalence (percent of time) of sound intensity (RMS, Root Mean Square) for left channel. (D) Prevalence (percent of time) of sound intensity (RMS, Root Mean Square) for Right channel. The intensity fluctuates slightly in level (the histogram is broad, focus at one RMS level, and is the same between channels). The difference between maximum and minimum intensity for BBN was 15 dB (both channels).

1. **Surf**

A


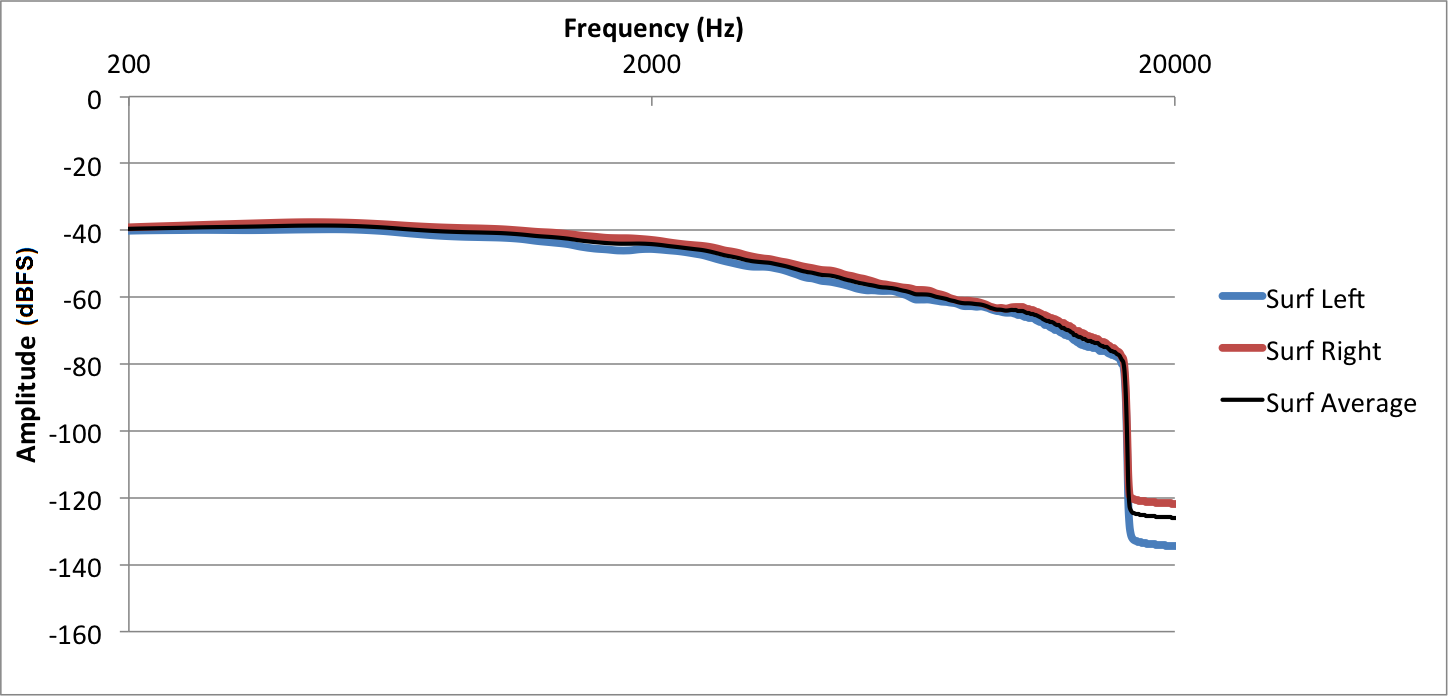


B


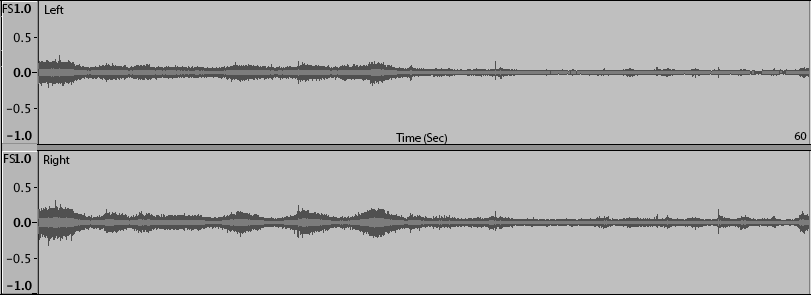


C


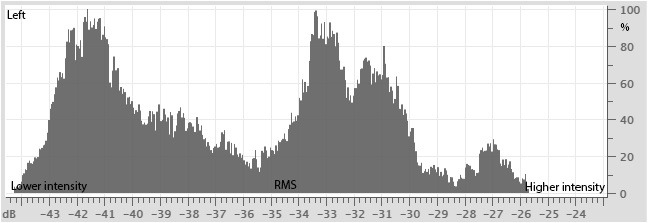


D


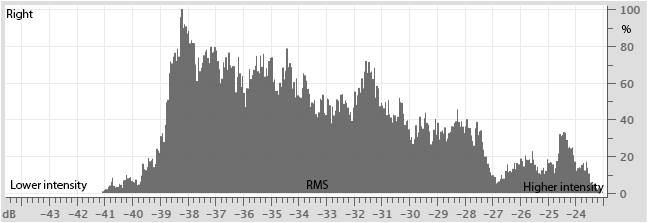


Acoustic characteristics of the intervention sound “Surf”. (A) Amplitude spectra (dB FS, decibels Full Scale) for each ear and average between ears. The spectrum recorded as a MP3 file is flat for frequencies below 2 kHz, very gently sloping between 2 and 16 kHz, rolling off at 16 kHz (as per MP3 psychoacoustic function). (B) Amplitude (FS, Full Scale ratio) over time (60 seconds) for each ear. The sound fluctuates with time. (C) Prevalence (percent of time) of sound intensity (RMS, Root Mean Square) for left channel. (D) Prevalence (percent of time) of sound intensity (RMS, Root Mean Square) for Right channel. The intensity fluctuates considerably in level (very broad histogram for left channel, with multiple (3) RMS level peaks; very broad histogram for right channel with no particular RMS level peaks). The intensity is slightly greater in the right channel (average RMS amplitude -33.3 dB) compared to the left channel (average RMS amplitude -36.1 dB). The difference between maximum and minimum intensity for Cicadas was 18.9 (left channel) and 18.2 (right channel).

1. **Cicadas**

A


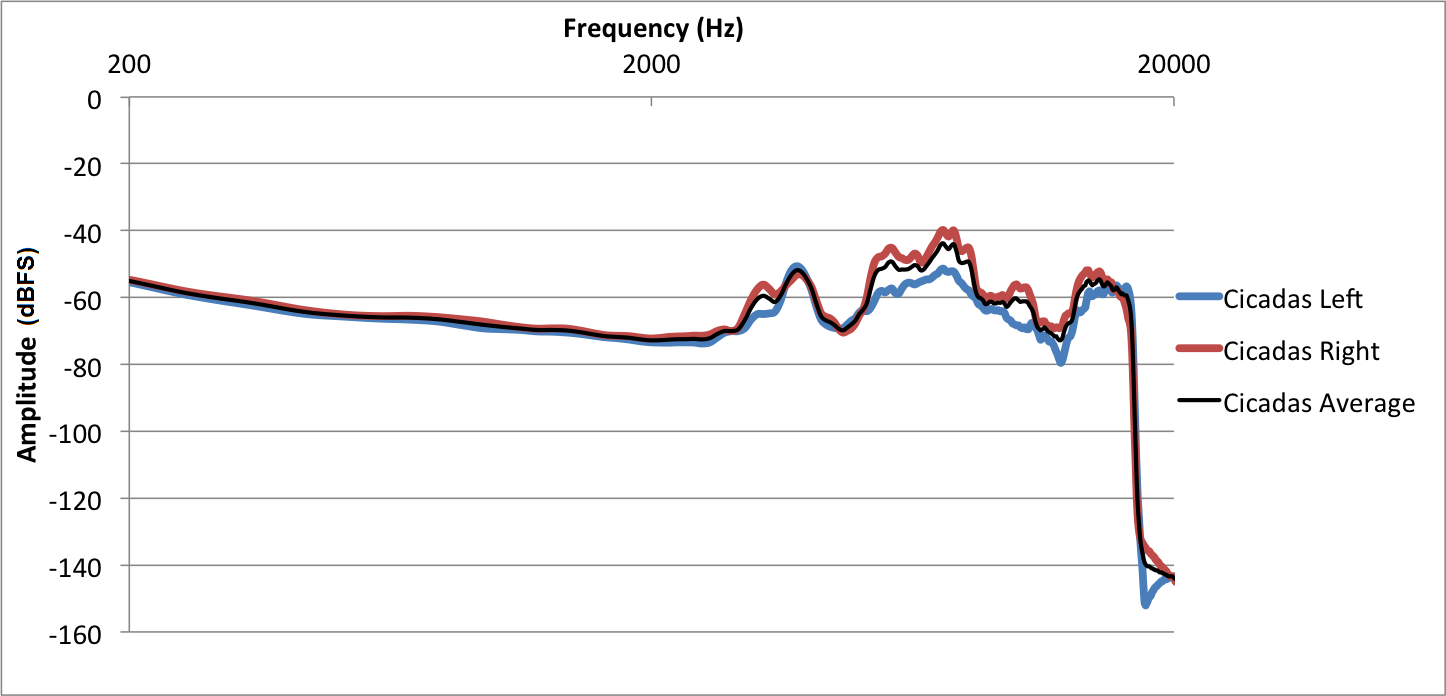


B


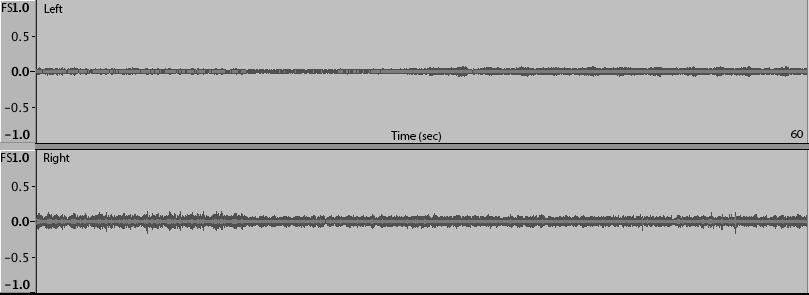


C


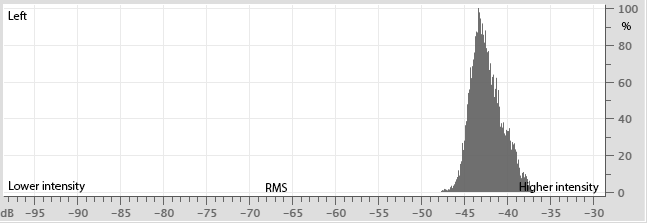


D


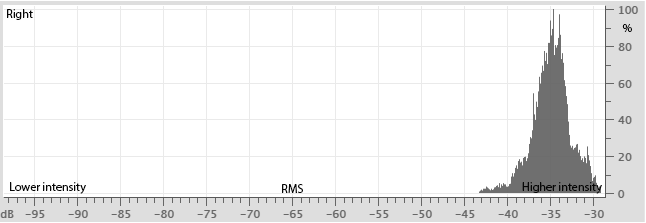


Acoustic characteristics of the intervention sound “Cicadas”. (A) Amplitude spectra (dB FS, decibels Full Scale) for each ear and average between ears. The spectrum recorded as a MP3 file is gently sloping for frequencies below 2 kHz, with multiple peaks between 2 and 16 kHz, rolling off at 16 kHz (as per MP3 psychoacoustic function). (B) Amplitude (FS, Full Scale ratio) over time (60 seconds) for each ear. The sound does not fluctuate with time. (C) Prevalence (percent of time) of sound intensity (RMS, Root Mean Square) for left channel. (D) Prevalence (percent of time) of sound intensity (RMS, Root Mean Square) for Right channel. The intensity does not fluctuate in level (the histogram is narrow, focus at one RMS level). The intensity is greater in the right ear (average RMS amplitude -34.9 dB) compared to the left ear (average RMS amplitude -42.4 dB). The difference between maximum and minimum intensity for Cicadas was 60.4 (left channel) and 69.1 (right channel).

1. **BBN**

A


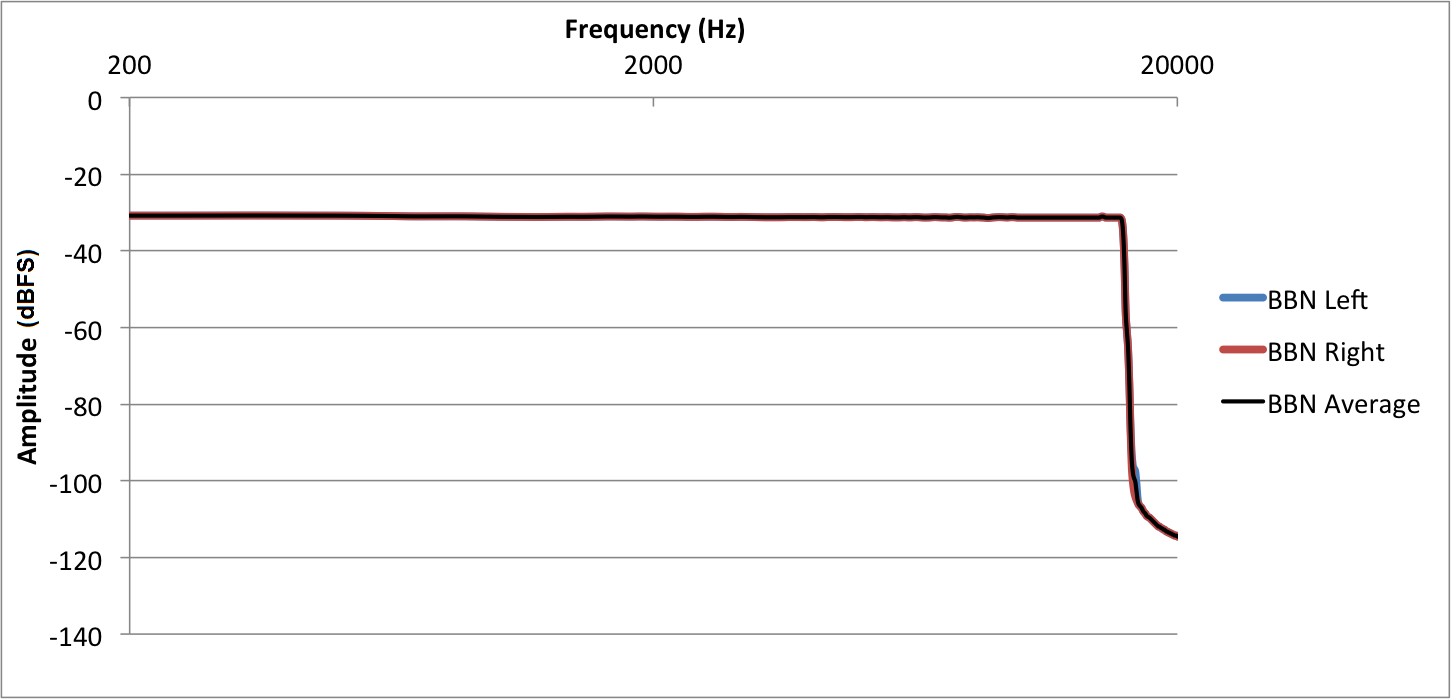


B


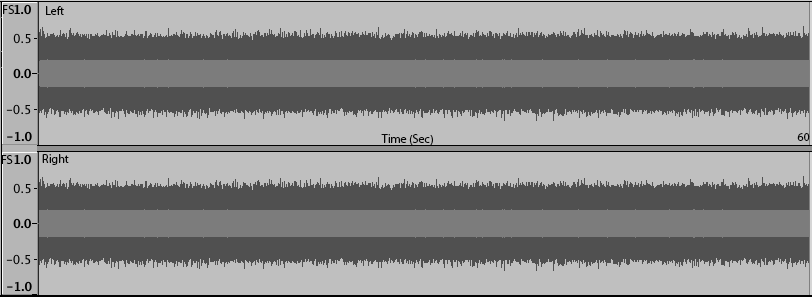


C


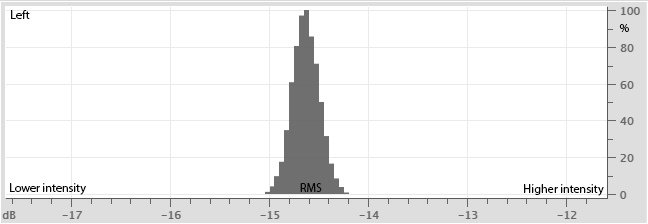


D

**
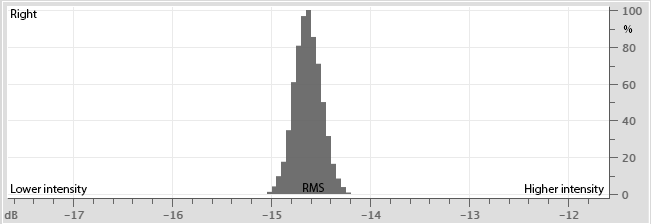
**

Acoustic characteristics of the intervention sound “BBN”. (A) Amplitude spectra (dB FS, decibels Full Scale) for each ear and average between ears. The spectrum recorded as a MP3 file is flat, rolling off at 16 kHz (as per MP3 psychoacoustic function). (B) Amplitude (FS, Full Scale ratio) over time (60 seconds) for each ear. The sound does not fluctuate with time. (C) Prevalence (percent of time) of sound intensity (RMS, Root Mean Square) for left channel. (D) Prevalence (percent of time) of sound intensity (RMS, Root Mean Square) for Right channel. As a constant noise the intensity does not fluctuate in level (the histogram is narrow, focus at one RMS level, and is the same between channels). The difference between maximum and minimum intensity for BBN was 15 dB (both channels).
