# Supplementary material for: A Mixed-Methods Trial of Broad Band Noise and Nature Sounds for Tinnitus Therapy: Group and Individual Responses Modeled under the Adaptation Level Theory of Tinnitus
Source: Front Aging Neurosci. 2017 Mar 9;9:44. doi: 10.3389/fnagi.2017.00044 (PMC5343046; doi:10.3389/fnagi.2017.00044)
Supplement: Supplementary file 2 [file DataSheet2.DOCX]

**Appendix B. Baseline characteristics of participants as measured at the start of the trial. ^a^Not fully maskable at level – terminated early for participant comfort.**

|  | Demographics |  | Tinnitus Characteristics | |  |  |  |  |
| --- | --- | --- | --- | --- | --- | --- | --- | --- |
| Participant | **Age** | **Gender** | **Duration (Yrs)** | **Loudness Rating (1-10)** | **Annoyance Rating (1-10)** | **Total TFI Score (weighted)** | **LLM** | **MML** |
| 1 | 38 | F | 6 | 6 | 5 | 36 | 7 | 4 |
| 2 | 62 | M | 30 | 6 | 6 | 32 | 13 | 17 |
| 3 | 65 | F | 40 | 6 | 5 | 51.2 | 0 | 6 |
| 4 | 62 | F | 17 | 6 | 7 | 46 | 0 | 0 |
| 5 | 67 | M | 20 | 7 | 6 | 60 | 2 | 6 |
| 7 | 58 | M | 10 | 7 | 6 | 63.2 | 8 | 6 |
| 8 | 65 | F | 20 | 4 | 3 | 24.8 | 0 | 6 |
| 9 | 66 | M | 10 | 7 | 5 | 24.8 | 8 | 7 |
| 10 | 65 | F | 18 | 8 | 9 | 44.8 | 12 | 20 |
| 11 | 57 | M | <1 | 8 | 3 | 76.8 | 1 | 8 |
| 12 | 61 | M | 45 | 7 | 5 | 44.4 | 1 | 3 |
| 13 | 55 | F | 10 | 7 | 6 | 36 | 3 | 30^a^ |
| 14 | 49 | M | 10 | 6 | 6 | 32.4 | 0 | 0 |
| 15 | 41 | M | 7 | 7 | 6 | 48.4 | 3 | 14 |
| 16 | 63 | M | <1 | 8 | 9 | 22.4 | 2 | 4 |
| 17 | 52 | M | <1 | 8 | 6 | 52 | 2 | 0 |
| 18 | 61 | F | 4 | 4 | 3 | 31.2 | 3 | 4 |

|  | Emotional/Psychological | | |  |  | Personality Traits | |  |  |
| --- | --- | --- | --- | --- | --- | --- | --- | --- | --- |
| Participant | **Positive Emotionality** | **Negative Emotionality** | **Anxiety** | **Depression** | **Stress** | **Stress Reaction** | **Social Closeness** | **Self Control** | **Alienation** |
| 1 | 28 | 17 | 2 | 0 | 3 | 6 | 6 | 17 | 0 |
| 2 | 41 | 10 | 1 | 1 | 0 | 4 | 2 | 12 | 2 |
| 3 | 34 | 13 | 2 | 1 | 1 | 6 | 5 | 13 | 3 |
| 4 | 37 | 11 | 0 | 0 | 0 | 3 | 8 | 12 | 0 |
| 5 | 26 | 16 | 14 | 10 | 13 | 10 | 4 | 17 | 1 |
| 7 | 28 | 30 | 10 | 10 | 23 | 13 | 9 | 17 | 1 |
| 8 | 33 | 11 | 0 | 2 | 3 | 5 | 7 | 10 | 0 |
| 9 | 36 | 12 | 0 | 0 | 4 | 2 | 2 | 14 | 4 |
| 10 | 41 | 15 | 0 | 0 | 7 | 10 | 6 | 13 | 0 |
| 11 | 32 | 19 | 8 | 2 | 8 | 2 | 7 | 17 | 0 |
| 12 | 30 | 17 | 3 | 2 | 4 | 5 | 6 | 17 | 2 |
| 13 | 44 | 14 | 0 | 4 | 5 | 3 | 8 | 16 | 0 |
| 14 | 40 | 21 | 2 | 10 | 16 | 8 | 2 | 10 | 2 |
| 15 | 24 | 21 | 3 | 6 | 13 | 12 | 5 | 9 | 3 |
| 16 | 43 | 27 | 18 | 0 | 8 | 8 | 1 | 15 | 2 |
| 17 | 31 | 15 | 15 | 0 | 4 | 11 | 8 | 10 | 2 |
| 18 | 33 | 9 | 4 | 3 | 6 | 10 | 9 | 18 | 0 |
