# Supplementary material for: A Mixed-Methods Trial of Broad Band Noise and Nature Sounds for Tinnitus Therapy: Group and Individual Responses Modeled under the Adaptation Level Theory of Tinnitus
Source: Front Aging Neurosci. 2017 Mar 9;9:44. doi: 10.3389/fnagi.2017.00044 (PMC5343046; doi:10.3389/fnagi.2017.00044)
Supplement: Supplementary file 3 [file DataSheet3.DOCX]

**Appendix C: Qualitative patient comments corresponding with related Results sections**

**3.2. Selection of Nature Sounds (Rain)**

*“The most pleasant, easier to listen to, happy to be inside when it is raining outside where its warm and dry”*

*“Selected rain in the end – tinnitus has various pitches so rain might be better for masking across frequencies & is pleasant”*

### 3.8. Intervention Outcomes: Qualitative Reports

**Hours & Environments of use**

*“Sometimes let it go on if reading, etc. at other times interrupted.”*

*“An hour a day... If sitting and in between patterns with knitting, keep looping”*

*“Have tried when walking dogs but traffic noise is too loud”*

*“Sometimes wear it to café for tea, lunch as well, general group asked once explained normal voice – could hear them fine, so it’s been quite good.”*

*“Have worn it while watching TV, did not find any interference.”*

**Early Termination of Trial**

*“The tinnitus became worse 4 weeks after use. During this time I was exposed to a high pitched noise in the lab, from a chiller, where I work on a daily basis (about 1 hour per day), and some of the time I could not wear hearing protection as I was having site meetings. This instantly made the tinnitus worse. Even having to wear earmuffs in lab after as a result, it shuts out ambience noise and focuses on tinnitus, so I’m more aware of it.”*

*“Unfortunately my tinnitus seems to have got worse lately, particularly at night. I have been waking up in the early hours and then having trouble getting back to sleep… The reason gave it away in the end, is because I started to wake up in the middle of night – tinnitus was there and trouble getting back to sleep again – never had that problem before so think it is because of the sound, but I am not sure whether it is the tinnitus that wakes me or because of the recent stress in my life. Anyhow I have decided to stop using the sound.”*

*“Woke up last night and it was horribly loud. It isn’t beneficial to continue.”*

**Effectiveness of intervention Sounds at 4 weeks**

Reduction in tinnitus:

*“More often than not it actually has reduced in level”*

*“White noise moved tinnitus back from when I started”*

*“It certainly does make a difference, I think the sound of tinnitus has changed, seems quieter – maybe because you are feeling more relaxed”*

*“I don’t have severe days as much – loudness might have levelled out”*

*“Not as harsh as it was, softened it”*

Exacerbation of tinnitus:

*“Last month have started annoying me, more stress because can hear it more.”*

*Attention on the tinnitus on it increased, so increased sensitivity to the tinnitus – first week was really intense, starting to really annoy.”*

*“No change in tinnitus while sound is playing, after stopped listening tinnitus was louder. Earmuffs after lawn mowing have the same effect – perhaps due to blockage of the ear. For example, I slept with ear plugs and tinnitus was louder in the morning but goes away. Sleep patterns have been good.”*

No change in tinnitus:

*“Tinnitus is more or less the same.”*

*“Do not think tinnitus has changed due to wearing the device.”*

*“Don’t think tinnitus greatly changes, can still hear tinnitus if you concentrate on it. Tinnitus – feels it’s created by stress – one day to another stress changes, so changes day to day.”*

**Effectiveness of intervention Sounds at 8 weeks**

*“No change in tinnitus. Never notice tinnitus when sound is playing, in a way its disappointment because really wanted it to work.”*

*“Don’t think tinnitus has changed over the last month, probably more aware of it because of having the sound in there but don’t think it has increased or decreased in intensity.”*

**Preference of Intervention Sound at 8 weeks**

Preference for Nature sounds:

*“Just more relaxing, other sound wasn’t necessarily as chilled. The Surf was more relaxing, natural environment. Don’t know if there was a huge improvement in tinnitus… Took you away from it [tinnitus] for a bit which I guess is the useful bit, distracts you from it for awhile.”*

*“I can let it go for 6-7 hours and can also have conversations with it, it was a refuge.”*

*“Found other sound [nature sound] was more restful to listen to over time, ups and down, overall sound was easier to get lost in it. Not sure if there was a huge difference between the two in terms of interacting with tinnitus.”*

Preference for BBN:

*“Think for the first sound started to notice a difference, this sound has had more of a beneficial effect because more aware of situations where I say tinnitus is different.”*

*“Prefer the white noise better, although like the rain, might be because sound is “boring” where the nature is interesting, so attend to aspects of sound.”*

*“Certainly white noise – for nature sounds there were other sounds in the background which shouldn’t have been there.”*

*“Rain was better personally, find it more pleasant to listen to and would like to use it more. Both did interact with noise in my head, white noise masks tinnitus better because of constant sound but both seem usable to me.”*

*“Sound [BBN] is wonderful. The last one had the rain which was lovely, wasn’t sure I would like white noise but did… I actually had it on and if I was doing something I wouldn’t take notice of the time and would have it on for more than hour… What it did compared to the rain was that I wasn’t dwelling on it, it wasn’t distracting… White noise is great there because it is constant… White noise better at blending with the tinnitus.”*

*“This sound [BBN] has been more in the background: the crickets intensified my awareness of the other sound. This has been a more level response [BBN] even after I have taken it off. Stress in work life is keeping me awake and once awake the tinnitus makes it difficult to fall asleep at the moment. When I wasn’t under stress the tinnitus was manageable. That sound [nature sound] was probably more relaxing and nice, has a visual association of a forest. Whereas plain noise [BBN] is just a sound and doesn’t have any such associations. But easier to have white noise and hear people, with cicadas had to keep it off to hear people at work.”*

Tolerability of BBN:

“*When you think of white noise think it is something you don’t want to listen to but was quite pleasant”*

*“When first heard white noise thought it would be unpleasant and off-putting, but not like that at all.”*

**Long-term use of sound device for tinnitus management**

*“There is no objection to listen to it in the long term, depends on whether there is any benefit – so far can’t say there is any change in my tinnitus. However I didn’t find it a burden to use it and am happy to continue.”*

**Quality of Intervention Sounds**

*“White noise – going from 4 to 5 in volume was a big jump, needed more of a smoother jump – the volume could have been recorded at a lower level to give a bigger range of adjustment.”*

*“Both sound quality were really good, don’t know how you would change the quality. Neither were intrusive or unpleasant”*

*“Not too bad if I don’t do it for too long.”*
